# Occupancy modeling and resampling overcomes low test sensitivity to produce accurate SARS-CoV-2 prevalence estimates

**DOI:** 10.1186/s12889-021-10609-y

**Published:** 2021-03-23

**Authors:** Jamie S. Sanderlin, Jessie D. Golding, Taylor Wilcox, Daniel H. Mason, Kevin S. McKelvey, Dean E. Pearson, Michael K. Schwartz

**Affiliations:** 1 USDA Forest Service, Rocky Mountain Research Station, 2500 S. Pine Knoll Dr., Flagstaff, AZ USA; 2 USDA Forest Service, National Genomics Center for Wildlife and Fish Conservation, Rocky Mountain Research Station, Missoula, MT USA; 3 USDA Forest Service, Rocky Mountain Research Station, Missoula, MT USA; 4 Division of Biological Sciences, University of Montana, Missoula, MT USA

**Keywords:** Occupancy modeling, Optimal sampling, Repeated sampling, Sampling strategies

## Abstract

**Background:**

We evaluated whether occupancy modeling, an approach developed for detecting rare wildlife species, could overcome inherent accuracy limitations associated with rapid disease tests to generate fast, accurate, and affordable SARS-CoV-2 prevalence estimates. Occupancy modeling uses repeated sampling to estimate probability of false negative results, like those linked to rapid tests, for generating unbiased prevalence estimates.

**Methods:**

We developed a simulation study to estimate SARS-CoV-2 prevalence using rapid, low-sensitivity, low-cost tests and slower, high-sensitivity, higher cost tests across a range of disease prevalence and sampling strategies.

**Results:**

Occupancy modeling overcame the low sensitivity of rapid tests to generate prevalence estimates comparable to more accurate, slower tests. Moreover, minimal repeated sampling was required to offset low test sensitivity at low disease prevalence (0.1%), when rapid testing is most critical for informing disease management.

**Conclusions:**

Occupancy modeling enables the use of rapid tests to provide accurate, affordable, real-time estimates of the prevalence of emerging infectious diseases like SARS-CoV-2.

**Supplementary Information:**

The online version contains supplementary material available at 10.1186/s12889-021-10609-y.

## Background

The emergence of severe acute respiratory syndrome coronavirus 2 (SARS-CoV-2) as a worldwide pandemic has demonstrated both the logistical challenge of effectively monitoring an emerging infectious disease and the enormous health and socio-economic costs associated with failing to meet this challenge. The ability to accurately track an emerging infectious disease within a population is critical for balancing direct health impacts of the disease against socio-economic impacts of community mitigation strategies for the disease through “shelter in place” rules [1, 2]. Effective community-level monitoring, particularly at low disease prevalence, is essential to inform disease management to both suppress the initial invasion and to maintain low prevalence thereafter. To date, attention has focused on inadequate testing access, test supply chain disruptions, and testing delays [3–5], but, as these problems are resolved, the clear challenge remaining lies in test efficacy and optimal sampling strategies needed to provide accurate community-level metrics of disease dynamics.

Effectively managing an emerging infectious disease requires logistically feasible, fast, and accurate community-level monitoring to inform real-time decisions about community mitigation actions [2, 6]. A primary hurdle to achieving accurate community-level monitoring is the tradeoff between speed and accuracy inherent in disease tests; rapid tests (e.g., antigen tests developed by Abbott [7]) are more error prone and therefore have a lower sensitivity for disease detection [8–10]. Historically, and throughout the current pandemic, disease testing has focused on individual testing, emphasizing accurate diagnosis of symptomatic individuals conducted with slower, higher sensitivity tests [11]. For SARS-CoV-2 and other emerging diseases, initial sampling tends to target the most severe cases, whereby moderate and mild cases are not sampled, leading to an underestimation of true community prevalence [4].

Novel modeling approaches developed for sampling rare animals in wildlife sciences hold great potential to address the inherent limitations of rapid tests [12]. Because many wildlife species of concern are rare and/or sparsely distributed, this discipline has spent decades developing tools to improve population estimation with imperfect detection [13–15]. Imperfect detection translates to false negative and/or false positive errors as it relates to disease prevalence. One such class of models is called occupancy models [16, 17]. Occupancy models address imperfect detection by collecting repeated samples (observations) of a species to statistically model sampling error rates and use this information to improve estimates of species’ presence/absence. Importantly, occupancy models can account for imperfect detection arising from biological processes and the effort to observe these processes. For example, occupancy modeling has been used in India to overcome issues of sampling for tigers (*Panthera tigris*) linked to their rarity, camouflage, stealth, and nocturnal behaviors [18]. Occupancy modeling has also been used to generate unbiased estimates of pathogen occurrence and prevalence in wildlife [19]. Importantly, due to more resource limitations in wildlife sciences compared to human health fields, wildlife researchers have developed tools for optimizing sampling designs [20] that can be adapted to generate efficacious and accurate sampling designs for estimating human disease prevalence.

Here we test whether occupancy modeling frameworks that account for imperfect detection from false negatives (test sensitivity) due to biological and observation processes could be used to address low test sensitivity in SARS-CoV-2 rapid tests. We focused on U.S. county-level estimates, although this approach may be applied at larger geographic or population scales. We specifically ask two questions: 1) “Can an occupancy modeling framework applied to rapid, low-sensitivity tests provide similar accuracy (e.g., less bias and greater precision) estimates of SARS-CoV-2 prevalence as higher sensitivity, more accurate tests?”; 2) “Given a fixed number of tests (due to laboratory capacity, fixed budget, or combination of these constraints), what is the best sampling strategy for deploying rapid tests to optimize accuracy while considering logistical and cost constraints?” We conducted a series of simulations to answer these questions by deploying two types of tests for SARS-CoV-2: a rapid, low sensitivity, cheaper test and a slower, high sensitivity, yet more expensive test. We examined a range of SARS-CoV-2 prevalence values. In addition, because we were interested in effective sampling strategies, we examined how the proportion of individuals initially sampled, number of repeat tests, and proportion of individuals with repeat tests affected prevalence estimates.

## Methods

In its origins, occupancy modeling relies on repeated sampling for the presence of an organism at a site [16, 17]. Here, the organism we wish to detect is SARS-CoV-2, and the place, or site, is the individual where the disease might be found. A site—in this case a person—is “occupied” if the organism (the virus) is present, and “unoccupied” otherwise; occupied and unoccupied are analogous to infected and uninfected. The occupancy modeling goal as applied here is to determine the proportion of sites (people) within the population (county) that are occupied (infected) given imperfect detection from false negatives (test sensitivity). Thus, occupancy modeling provides an appropriate statistical design for estimating disease prevalence.

### Model

We used a Bayesian hierarchical occupancy model [21], which includes biological and observation processes. The following describes the variables used to model each process.

#### Biological process

Following a single-season occupancy model [16, 17], where *I* represents infected and *U* represents uninfected, we modeled true SARS-CoV-2 presence in an individual (*j*) as a latent (not directly observed) Bernoulli variable (*z*_j_) with probability of success as the average infection probability within individuals in the community (*ψ*_I_):

1 $$ {z}_j\sim Bernoulli\ \left({\Psi}_I\right). $$

Number of infected individuals (*N*_I_) and uninfected individuals (*N*_U_) can be estimated as derived parameters, and *N*_POP_ (number of individuals in the population) is known:

2 $$ {N}_I={\sum}_{j=1}^{N_{POP}}{z}_j, $$

and *N*_*U*_ = *N*_*POP*_ − *N*_*I*_.

#### Observational process

We modeled the observation process as a simple random sample (independent of symptoms) with imperfect detection from false negatives (low test sensitivity) as a zero-inflated binomial process. In occupancy modeling, there is a formal requirement for closure at the site level, i.e., with disease prevalence, each sampled person in the population must remain either infected or uninfected throughout the sampling period. Test sensitivity is estimated through repeated independent samples at a site. Individuals sampled (i.e., sites) would therefore have multiple tests (*N*_sample,j_) taken at a single ‘visit’ during the sampling period. This is difficult to achieve with Reverse Transcription quantitative PCR (RT-qPCR)-based tests but is amenable to rapid and inexpensive tests such as antigen tests developed by Abbott [7] or other similar tests. We modeled infected individual detection (*y*_j_) as a binomial random variable, with number of binomial trials represented as number of repeat tests per sampled individual (*N*_sample,j_) and success of those trials as an unknown probability (*mu*_j_), dependent on true SARS-CoV-2 presence in an individual (*z*_j_) and test sensitivity (*p*_*test*_) (*mu*_*j*_ = *z*_*j*_ × *p*_*test*_):

3 $$ {y}_j\sim Binomial\ \left({N}_{sample,j},{mu}_j\right). $$

In this case, infected individual detections (*y*_j_) and number of repeat tests per individual (*N*_sample,j_) represent known real world data from a simple random sample of a hypothetical county with SARS-CoV-2.

### Simulations

To evaluate multiple biological and observational process scenarios, we conducted a series of simulations that varied values within both processes. We used information from literature (if available) to support selection of low, medium, and high levels for each parameter (Additional file 1). We used the median U.S. county population size of 25,000 (Census Bureau 2019) across all simulations to represent population size (*N*_pop_) to reflect realistic SARS-CoV-2 U.S. sampling scenarios. To account for SARS-CoV-2 prevalence affecting test sensitivity, we examined simulations with three values of very low (0.001), low (0.01), and moderate (0.1) prevalence (*ψ*_I_). Within the observation process, we modeled deployment of two SARS-CoV-2 test types by modeling two test sensitivity (*p*_*test*_) values based on known test sensitivities (Additional file 1): low (0.30) and high (0.78).

In addition, because we were interested in optimal sampling strategies (question 2), we modified total number of tests per individual (*N*_sample,j_) by varying proportion of individuals initially sampled, number of repeat tests per individual, and proportion of individuals with repeat tests. We used three values for proportion of individuals initially sampled to represent low (0.001), medium (0.01), and high (0.05) proportions within the county. Because occupancy modeling relies on repeated testing, we modeled sampling with a single repeat test (2 tests total) or 4 repeat tests (5 tests per individual total). We also varied proportion of individuals that were repeatedly sampled to represent 10, 50 and 100% of the sampled individuals repeat tested to account for the potential that some individuals initially sampled are unwilling to be resampled in a single visit.

The combination of all parameter values described above resulted in 108 unique simulation scenarios (Additional file 1), which were created with program R [22]. To create a simulation scenario, we simulated a population (25,000) with occupied and unoccupied individuals (Eq. 1) with one of the three prevalence values. We then simulated sampling that population with imperfect detection from false negatives (test sensitivity; Eq. 3) and varied observation process parameters described above (proportion of individuals initially sampled, number of repeat tests, and proportion of individuals that were repeat sampled). Next, we used observed data for a single scenario as an input in the Bayesian hierarchical model (Eq. 1–3) Markov chain Monte Carlo (MCMC) process, ran the model in JAGS [23] using the *rjags*, *jagsUI* [24], and *coda* [25] packages, to obtain posterior estimates of SARS-CoV-2 prevalence (*ψ*_I_) and test sensitivity (*p*_*test*_). That was for one replicate of the simulation-estimation process for one scenario. We used 100 replicates of the simulation-estimation process for each simulation scenario. For each of the 100 simulation-estimation replicates, we used independent, non-informative priors for *ψ*_I_ and *p*_test_ and ran three parallel chains (length = 10,000 iterations, burn-in = 1000 iterations, no thinning) to estimate the posterior distribution median of model parameters and 95% Bayesian credible intervals (BCI) for each replicate. We assessed model convergence by using $\hat{R}$ < 1.1 [26]. We then compared true values we used to generate the biological and sampling processes to estimated prevalence (*ψ*_I_) and test sensitivity (*p*_*test*_) for each replicate of the simulation-estimation process over all simulation scenarios.

For all simulations, we assumed the population was closed to movement during the sampling time frame and each individual was available for sampling in the county. We also assumed disease state (i.e., occupied or unoccupied) did not change during the sample period. These conditions equate to a short sampling time window (point prevalence).

### Evaluating simulations for occupancy modeling (question 1)

To evaluate if an occupancy modeling framework with rapid tests could provide accurate SARS-CoV-2 prevalence estimates, we examined relative root mean square error (RRMSE) for prevalence (*ψ*_I_) and test sensitivity (*p*_*test*_). RRMSE, or accuracy, is the combination of bias and precision defined as:

4 $$ RRMSE=\frac{\sqrt{\left(1/r\right){\sum}_{i=1}^n{\left({\hat{\theta}}_i-{\theta}_i\right)}^2}}{\overline{\theta}}, $$

where *r* is number of replicates, ${\hat{\theta}}_i$ is the estimated parameter (posterior median) at replicate *i*, *θ*_*i*_ is the true parameter value at replicate *i*, and $\overline{\theta}$ is the mean of the true parameter values over all replicates. We also calculated relative bias (RBIAS), percent coverage, and Bayesian Credible Interval (BCI) length (Additional file 2, Additional file 3).

### Evaluating simulations for optimal sampling strategies (question 2)

To evaluate optimal sampling strategies given fixed resources (i.e., number of tests available, fixed budget) at the county level, we used a constrained optimization framework [27] consisting of three components: (i) decision variables (proportion of individuals initially sampled, number of repeat tests per individual, proportion of individuals with repeat tests), (ii) objective function (minimize SARS-CoV-2 prevalence RRMSE), and (iii) constraints (total number of samples represented as a cost). We illustrate this framework using a cost constraint, but this framework can also include a time constraint, as quicker results could influence individual behavior and contribute to slowing disease spread [6]. The cost function was:

5 $$ C={C}_s\times {N}_{sample}, $$

where *C* was total cost; *C*_s_ was per sample cost for collecting sample, sample storage, sample preparation, and test materials; and *N*_sample_ was total number of samples (sum of samples from initially sampled individuals within the county and from all repeated tests for a subset of individuals). For *p*_test_ of 0.3 associated with a rapid test, *C*_s_ was $5 [28]. For *p*_test_ of 0.78 associated with a RT-qPCR test, *C*_s_ was $100. We expect laboratory costs to vary by county and laboratory technician and collection staff salary, and thus present a general cost function to illustrate a framework to evaluate accuracy and associated costs with decision variables for different test types. We also recognize that start-up costs for laboratories may be substantial, thus we assume counties will utilize laboratories that already have necessary equipment and technical expertise.

Given our RRMSE prevalence simulation values for each combination of decision variables, our objective function was to minimize prevalence RRMSE subject to constraints:

$$ C={C}_0+{C}_s\times {N}_{sample}\le d, $$

$$ RRMSE\le e. $$

We constrained total cost below *d* to evaluate a range of sampling strategies given a fixed number of tests available (due to laboratory capacity, fixed budget, or combination of these constraints) and RRMSE below *e* to represent a desired amount of prevalence accuracy. We demonstrated the optimization process graphically: optimal sampling strategy was determined by examining where accuracy (RRMSE) asymptotes given costs (i.e., there is marginal gain for additional sampling) before cost constraints.

## Results

Overall, we found that occupancy modeling in conjunction with resampling strategies can overcome low test sensitivity associated with rapid tests to provide accurate SARS-CoV-2 prevalence estimates comparable to those of more accurate but slower tests. In addition, we identified optimal sampling strategies using cost constraints across all prevalence levels. The specific results of each are discussed below for simulation data (Additional file 4).

### Evaluating simulations for occupancy modeling (question 1)

#### Accounting for biological and observation processes

**Fig. 1:**
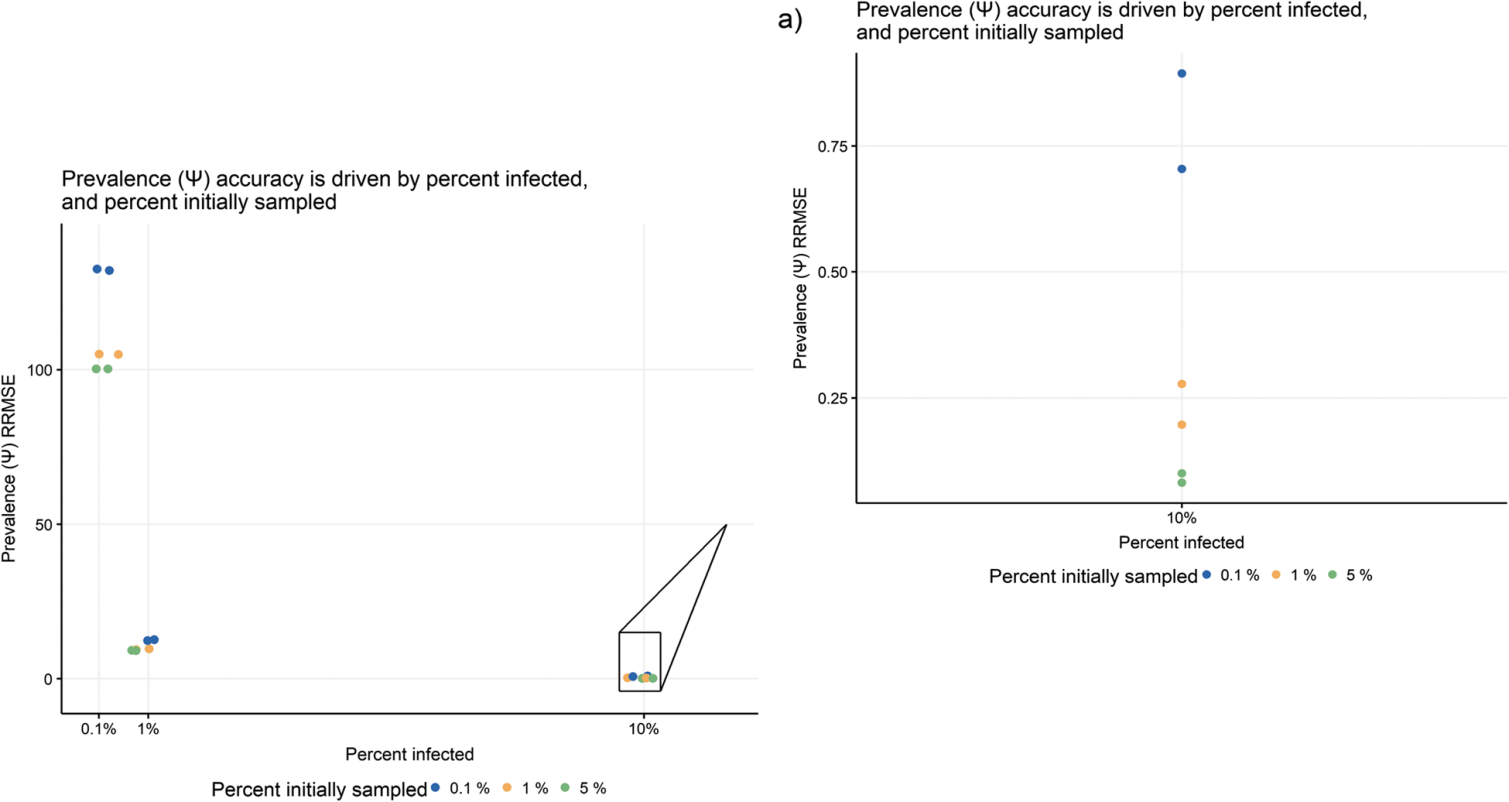
Prevalence accuracy as a function of percent of the population infected. Accuracy (relative root mean square error) of prevalence (*Ψ*) as a function of true prevalence, or percent of the population infected, from simulation scenarios of 100% of the initial sample with 5 repeat tests. The inset a) is for simulation scenarios with 10% of the population infected (true prevalence)

**Fig. 2:**
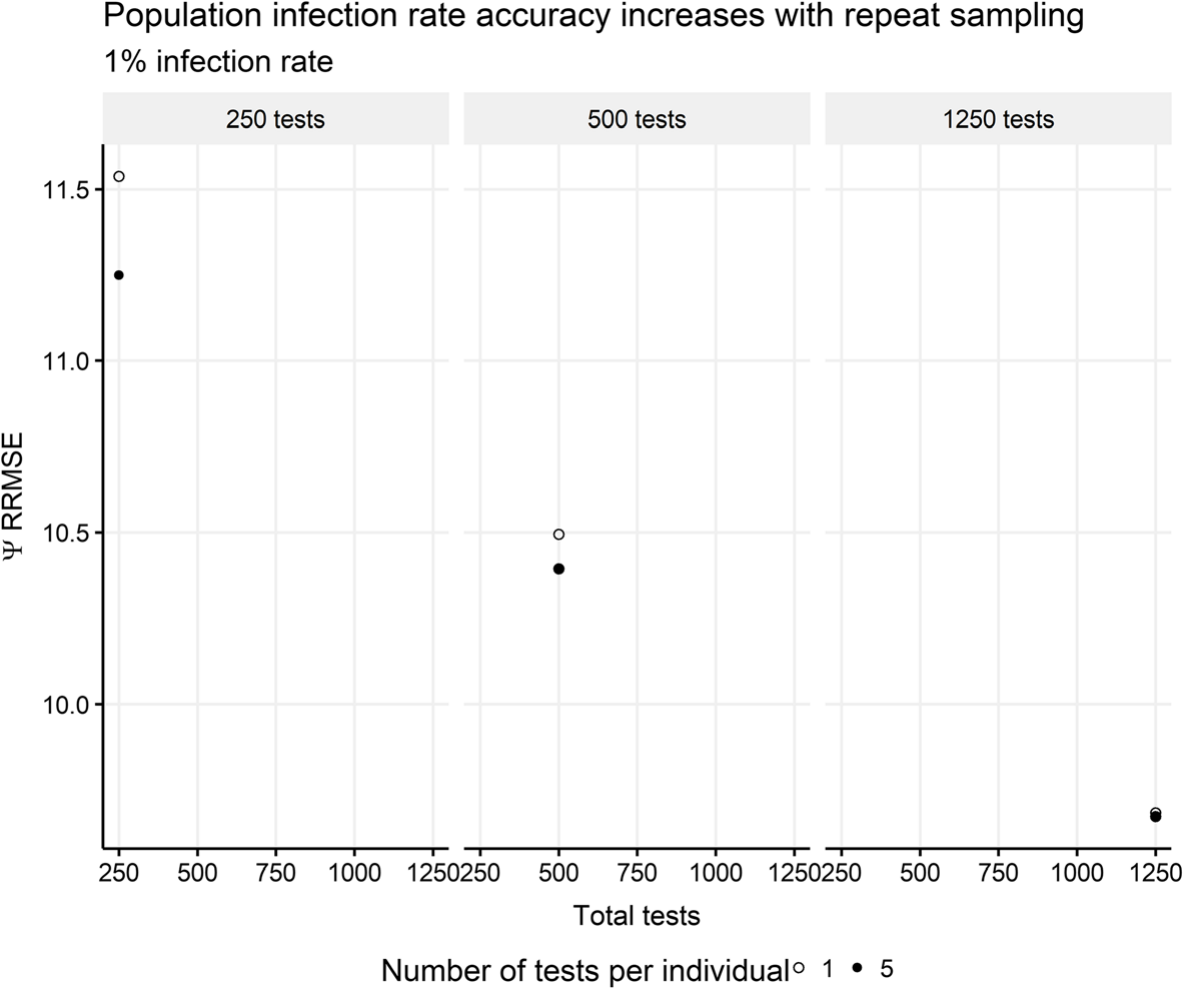
Prevalence accuracy as a function of total tests. Accuracy (relative root mean square error) of prevalence (*Ψ*) as a function of total tests from simulation scenarios of 1% infection rate and test sensitivity of 0.3. Note that the percent of the population initially sampled is not the same with one test (no repeat) versus 5 tests (4 repeats)

**Fig. 3:**
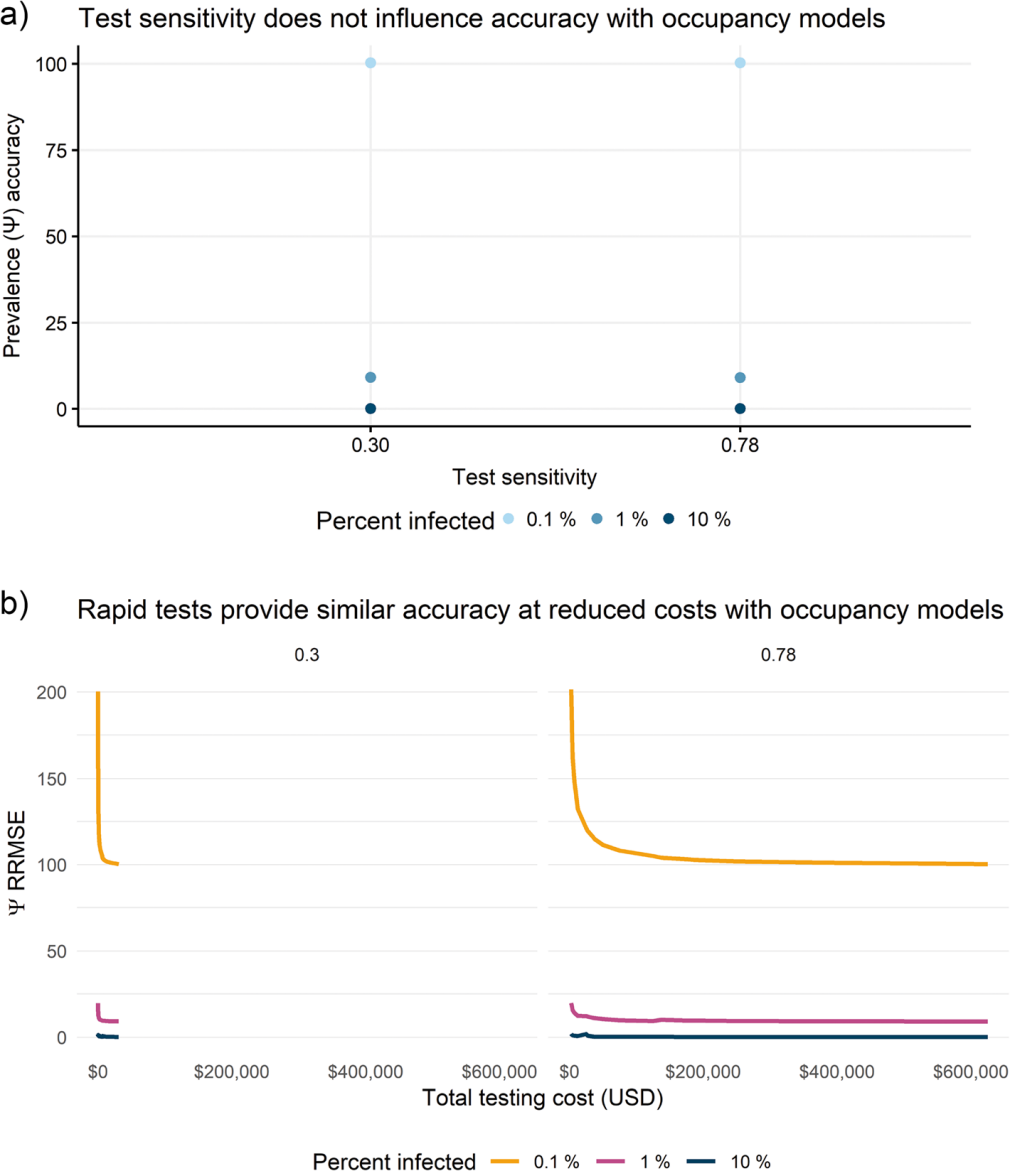
Prevalence accuracy as a function of true test sensitivity and costs. Accuracy (relative root mean square error) of prevalence (*Ψ*) as a function of a) true test sensitivity from simulation scenarios of 5% of the population initially sampled and 100% of that sample with 5 repeat tests, and b) costs associated with the two test types

Relative bias and accuracy (RRMSE) of SARS-CoV-2 prevalence were influenced by both true prevalence (biological process) and sampling strategy (observation process). Estimates were influenced most by prevalence magnitude, followed by sampling strategy (Figs. 1-3, Additional file 2). Not surprisingly, overall, SARS-CoV-2 prevalence accuracy was lower and more variable among sampling strategies when the disease was extremely rare (true prevalence 0.001). Accuracy improved as prevalence increased and/or a greater proportion of the population was sampled. With true prevalence of 0.01 and 0.1, accuracy increases (RRMSE decreases) were smaller with increases in the proportion of a population initially sampled.

#### Improvement with repeated testing

More repeat tests greatly improved SARS-CoV-2 prevalence accuracy (RRMSE) estimates, especially with fewer total tests (Fig. 2). For example, when a county had 1% prevalence and 250 tests to allocate, if 50 people get 5 repeat tests the RRMSE was reduced by 2.2% compared to a scenario with 250 people that got a single test.

#### Occupancy modeling overcomes lower test sensitivity of rapid tests

Occupancy modeling overcame the lower test sensitivity of rapid tests compared with high-accuracy tests, i.e., SARS-CoV-2 prevalence accuracy was similar for low-sensitivity rapid and higher-sensitivity, slower tests for all SARS-CoV-2 prevalence levels and sampling strategies when occupancy models were applied (Fig. 3, Additional file 2). For example, with 5% of the population initially sampled and 100% of that sample with 5 repeat tests, we found prevalence RRMSE was similar for prevalence of 0.1% (RRMSE = 100.29 for *p*_test_ = 0.3, RRMSE = 100.30 for *p*_test_ = 0.78; Fig. 3a). However, rapid tests (*p*_test_ = 0.3) provide similar accuracy at reduced costs with occupancy models (Fig. 3b).

### Evaluating simulations for optimal sampling strategies (question 2)

**Fig. 4:**
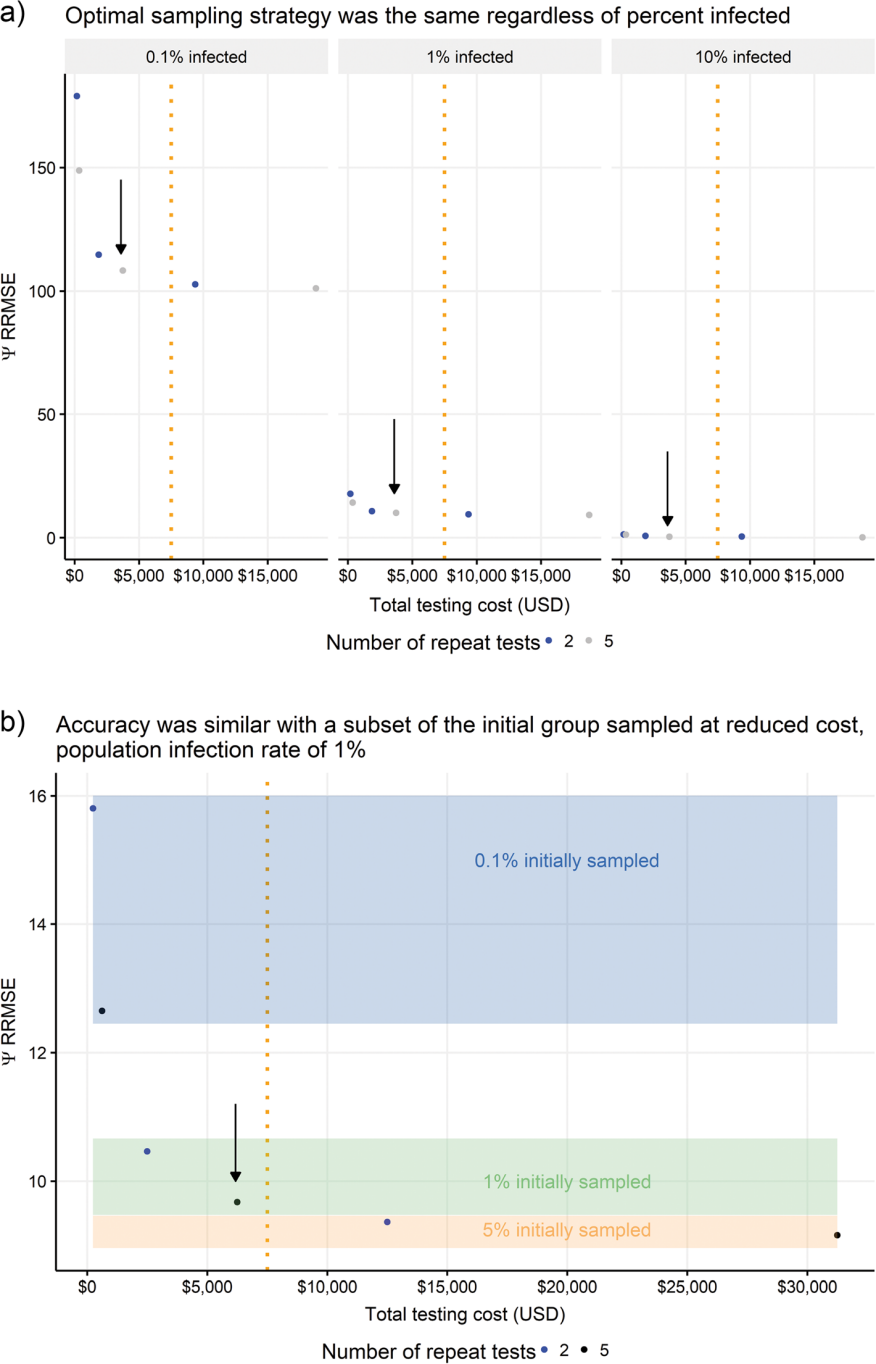
Optimal sampling designs with prevalence accuracy as a function of costs. Accuracy (relative root mean square error) of prevalence (*Ψ*) as a function of costs (USD) using arbitrary cost constraint (dotted vertical line) of $7500 USD for a) a subset of the simulation scenarios with the true test sensitivity of 0.3 and 50% of the initially sampled population with repeat tests; b) a subset of the simulation scenarios with the true test sensitivity of 0.3, true population infection rate (true prevalence) of 1, and 100% of the initially sampled population with repeat tests. Optimal designs are indicated within the figures with arrows

Across all disease prevalence levels, the optimal (lowest RRMSE relative to cost constraints) sampling strategy for a fixed proportion of the initially sampled population with repeat tests was: i) with 1% of population initially sampled, and ii) sampling occurring five times per individual with repeat tests (1 initial test and 4 repeat tests) (Fig. 4, Supplementary Figs. 10 and 11 in Additional file 2). Our simulation scenarios considered resampling a subset of the initial group sampled, in addition to 100% of the initial group sampled. Similar accuracy can be achieved with only a subset of the initial group sampled, for reduced overall costs (Fig. 4, Additional file 2).

## Discussion

Mitigating the impacts of emerging infectious disease like SARS-CoV-2 requires rapid testing to generate real-time data for informed disease management. We demonstrate how occupancy modeling can overcome low test sensitivity with rapid COVID-19 surveillance schemes to generate accurate (high-precision, low-bias) SARS-CoV-2 prevalence estimates. Moreover, the ability of this approach to offset test sensitivity with rapid tests at low disease prevalence is crucial because decisive disease management actions are most critical at low prevalence levels such as during disease onset and resurgence following control. Rapid tests are also inexpensive and logistically easy to administer, enabling the additional sampling effort requisite for resampling designs [10] with quick turn-around times. While our modeling efforts targeted the challenge associated with low sensitivity tests, occupancy modeling holds potential to address other rapid testing limitations for improved disease management.

To further advance rapid testing designs for disease monitoring, other shortfalls will also need to be addressed. In addition to low test sensitivity or false negative results, rapid tests also produce false positive results, or low specificity [29]. False positive results can impact patient risk and costs with unnecessary sequestration or even worse – uninfected individuals being assigned to COVID-19 hospital wards where they may become infected [29]. We did not account for false positives. However, similar methods exist to account for false positive detections [30, 31]. Moreover, more sophisticated approaches can be applied to refine estimates under complex, real-world conditions that account for symptoms at the time of testing and incorporate more underlying biological processes, including an instantaneous model using stratified sampling of states (symptomatic vs. asymptomatic) with no transitions among states using single-season, multi-state occupancy models with state uncertainty [32, 33], and Hidden Markov models with SIR (susceptible infected recovered) models using state transitions both in discrete and continuous time [34]. An alternative to stratified sampling could include collection of site-level covariates to improve detection at sample collection time (i.e., symptomatic status, symptom start date) an approach common in occupancy models in the wildlife literature [18, 19]. We stress that the approach we present is designed to assess disease prevalence across a population. It is not intended for determining infection status of individuals (although see Additional file 5). Nor is it appropriate for circumstances where institutions seek to create an infection-free group. Such objectives require high test sensitivity at the individual level. Nonetheless, information from such individual-oriented testing could be incorporated into prevalence estimates in models like the one we introduced.

## Conclusions

For emerging infectious diseases like COVID-19, rapid testing is essential for generating the real-time disease monitoring data that is required to inform disease management actions and minimize human health impacts [2, 6]. Resolving this sampling challenge is essential, especially as winter arrives in the northern hemisphere where onset of additional respiratory diseases with similar symptomology (e.g., rhinoviruses, seasonal coronaviruses, influenza) will confound SARS-CoV-2 detection. We demonstrate how occupancy modeling can help to overcome low test sensitivity to produce accurate disease prevalence estimates for real-time, informed decision making, even at low disease prevalence levels when decisive action is most meaningful. We also show the optimal sampling strategy in combination with occupancy modeling will be equally effective for community-level inference at different points in the course of an epidemic. Finally, we demonstrate that additional testing beyond the optimal sampling strategy in combination with occupancy modeling will not substantively improve prevalence estimates, allowing funds to be directed to the most pertinent disease mitigation measures.

## Supplementary Information

**Additional file 1.** Simulation study parameter combinations. Table of simulation study parameter combinations for evaluating the effectiveness of different sampling and biological parameters.

**Additional file 2.** Simulation summary. Document describing the simulation summary for relative root mean square error, relative bias, percent coverage, and Bayesian Credible Interval length.

**Additional file 3.** Simulation code. R code of simulations (GitHub: https://github.com/jamiesanderlin/Sanderlin-et-al-occupancy-modeling-and-SARS-CoV-2-prevalence).

**Additional file 4.** Simulation data. Simulation data summarized by simulation parameter combination. These data were used for all plots presented within the paper.

**Additional file 5.** Individual-level inference from occupancy model results. Document describing individual-level inference from occupancy model results.

## Data Availability

All data generated or analyzed during this study are included in this published article [and its supplementary information files].

## Abbreviations

BCI: Bayesian Credible Interval
MCMC: Markov Chain Monte Carlo
RBIAS: relative bias
RRMSE: relative root mean square error
RT-qPCR: Reverse Transcription quantitative Polymerase Chain Reaction
SARS-CoV-2: severe acute respiratory syndrome coronavirus 2
SIR: susceptible infected recovered
