# Supplementary material for: Occupancy modeling and resampling overcomes low test sensitivity to produce accurate SARS-CoV-2 prevalence estimates
Source: BMC Public Health. 2021 Mar 23;21:577. doi: 10.1186/s12889-021-10609-y (PMC7986646; doi:10.1186/s12889-021-10609-y)
Supplement: Supplementary file 1 — Additional file 1. Simulation study parameter combinations. Table of simulation study parameter combinations for evaluating the effectiveness of different sampling and biological parameters. [file 12889_2021_10609_MOESM1_ESM.pdf]

Additional File 1. Simulation study parameter combinations. Simulation study parameter combinations for evaluating the effectiveness of different sampling and biological parameters.

Parameter	Type	Low	Medium	High	Justification
Number of repeat tests	Sampling	2		5	Arbitrary
Proportion of sampled individuals with repeat tests	Sampling	0.10	0.50	1.0	Arbitrary
Proportion of sampled individuals	Sampling	0.001	0.01	0.05	U.S. average daily testing rate ~0.1% of population/day in May 2020 (1,2), Harvard "Massive Scale Testing" plan calls for testing 2 - 6% of population per day (3)
Test sensitivity (detection probability)	Sampling	0.30		0.78	Reports in the literature are highly variable (4-16). A literature review by (16) found that sensitivity for nasal swabs averaged 73.3% (95% CI 68.1–78.0%) when using RT-qPCR. There are, however, reasons to believe that these values may be biased high when compared to

samples taken from random and therefore primarily non-symptomatic patients. Some proportion of the sample will have just contracted the disease and will likely have low viral loads in their nasal passages. Similarly, some proportion of the population will be asymptomatic: we know little about this group but can assume that viral loads in their nasal passages will likely be lower than those that are either presymptomatic or symptomatic. If this is the case, tests on asymptomatic patients would be expected to have lower sensitivity than indicated by published results. We assume that there would be a group of recently infected people in our sample, entirely asymptomatic, who would be sick, but would seldom test positive (17).

Probability of being infected (ψ_I)	Biological	0.001	0.01	0.10	Total confirmed positive overall in the US is ~2.3% (7,894,768 U.S. cases confirmed positive (18), 330,455,538 U.S. population (19)). Range of values were selected to be relevant for surveillance.

References

1. Coronavirus (COVID-19) Testing [Internet]. [cited 2020 May 15]. Available from: https://ourworldindata.org/coronavirus-testing#the-scale-of-testing-compared-to-the-scale-of-the-outbreak

2. The COVID Tracking Project [Internet]. [cited 2020 May 15]. Available from: https://covidtracking.com/data

3. Bryant, JamesAllen D, Block S, Cohen J, Eckersley P, Eifler M, Gostin L, et al. Roadmap to pandemic resilience [Internet]. Edmond J. Safra Center for Ethics, Harvard University; 2020. p. 56. Available from: https://ethics.harvard.edu/covid-roadmap

4. Scohy A, Anantharajah A, Bodéus M, Kabamba-Mukadi B, Verroken A, Rodriguez-Villalobos H. Low performance of rapid antigen detection test as frontline testing for COVID-19 diagnosis. J Clin Virol [Internet]. 2020;129(May):104455. Available from: https://doi.org/10.1016/j.jcv.2020.104455

5. Mak GC, Cheng PK, Lau SS, Wong KK, Lau CS, Lam ET, et al. Evaluation of rapid antigen test for detection of SARS-CoV-2 virus. J Clin Virol [Internet]. 2020;129(June):104500. Available from: https://doi.org/10.1016/j.jcv.2020.104500

6. Linares M, Pérez-Tanoira R, Carrero A, Romanyk J, Pérez-García F, Gómez-Herruz P, et al. Panbio antigen rapid test is reliable to diagnose SARS-CoV-2 infection in the first 7 days after the onset of symptoms. J Clin Virol. 2020;133(September):3–6.

7. Xie X, Zhong Z, Zhao W, Zheng C, Wang F, Liu J. Chest CT for typical coronavirus disease 2019 (COVID-19) pneumonia: relationship to negative RT-PCR testing.

Radiology. 2020;296(2):E41–5.

8. Arevalo-Rodriguez I, Buitrago-Garcia D, Simancas-Racines D, Zambrano-Achig P, del Campo R, Ciapponi A, et al. False-Negative Results of Initial Rt-Pcr Assays for Covid-19: a Systematic Review. 2020;1–33.

9. Williams TC, Wastnedge E, McAllister G, Bhatia R, Cuschieri K, Kefala K, et al. Sensitivity of RT-PCR testing of upper respiratory tract samples for SARS-CoV-2 in hospitalised patients: a retrospective cohort study. medRxiv.

10. Luo L, Liu D, Wang Z, Chen P, Mao C. Modes of contact and risk of transmission in COVID-19 : a prospective cohort study 4 950 close contact persons in Guangzhou of China. Lancet.

11. Fang Y, Nie Y, Penny M. Transmission dynamics of the COVID-19 outbreak and effectiveness of government interventions: A data-driven analysis. J Med Virol. 2020;92(6):645–59.

12. Meyerson NR, Yang Q, Clark SK, Paige CL, Fattor WT, Gilchrist AR, et al. A community-deployable SARS-CoV-2 screening test using raw saliva with 45 minutes sample-to-results turnaround. medRxiv [Internet]. 2020;2020.07.16.20150250. Available from: https://doi.org/10.1101/2020.07.16.20150250

13. Chan JF-W, Yip CC-Y, To KK-W, Tang TH-C, Wong SC, Leung K-H, et al. Improved molecular diagnosis of COVID-19 by the novel, highly sensitive and specifice COVID-19-RdRp/Hel real-time reverse transcription-PCR assay validated in vitro and with clinical specimens. J Clin Microbiol. 2020;(April):1–10.

14. Long C, Xu H, Shen Q, Zhang X, Fan B, Wang C, et al. Diagnosis of the Coronavirus disease (COVID-19): rRT-PCR or CT? Eur J Radiol [Internet]. 2020;126(March):108961. Available from: https://doi.org/10.1016/j.ejrad.2020.108961

15. Woloshin S, Patel N, Kesselheim AS. False negative tests for SARS-CoV-2 infection - challenges and implications. N Engl J Med [Internet]. 2020;38(1):1–2. Available from: nejm.org

16. Böger B, Fachi MM, Vilhena RO, Cobre AF, Tonin FS, Pontarolo R. Systematic review with meta-analysis of the accuracy of diagnostic tests for COVID-19. Am J Infect Control. 2020;000.

17. Larremore DB, Wilder B, Lester E, Shehata S, Burke JM, Hay JA, et al. Test sensitivity is secondary to frequency and turnaround time for COVID-19 surveillance. medRxiv [Internet]. 2020;2020.06.22.20136309. Available from: https://www.medrxiv.org/content/10.1101/2020.06.22.20136309v2%0Ahttps://www.medrxiv.org/content/10.1101/2020.06.22.20136309v2.abstract%0Ahttps://www.medrxiv.org/content/10.1101/2020.06.22.20136309v2%0Ahttps://www.medrxiv.org/content/10.1101/2020.06.22.201

18. CDC COVID Data Tracker [Internet]. [cited 2020 Oct 16]. Available from: https://covid.cdc.gov/covid-data-tracker/#trends_dailytrendscases

19. U.S. and World Population Clock [Internet]. [cited 2020 Oct 16]. Available from: https://www.census.gov/popclock/
