# Supplementary material for: Occupancy modeling and resampling overcomes low test sensitivity to produce accurate SARS-CoV-2 prevalence estimates
Source: BMC Public Health. 2021 Mar 23;21:577. doi: 10.1186/s12889-021-10609-y (PMC7986646; doi:10.1186/s12889-021-10609-y)
Supplement: Supplementary file 2 — Additional file 2. Simulation summary. Document describing the simulation summary for relative root mean square error, relative bias, percent coverage, and Bayesian Credible Interval length. [file 12889_2021_10609_MOESM2_ESM.pdf]

Additional file 2. Simulation summary. Simulation summary for relative root mean square error, relative bias, percent coverage, Bayesian Credible Interval length, and optimization

As with any monitoring program designed to evaluate trends, standardized sampling designs that can achieve sufficient power to meet objectives will contribute to more robust monitoring data sets (1). Our simulation study was designed to evaluate the level of inference for disease prevalence at different biological and sampling scenarios, which is important information to determine if sufficient power is available to meet objectives for disease mitigation strategies. We evaluated bias, percent coverage, and precision with our simulation scenarios (described below) for the parameters of prevalence and test sensitivity, in addition to accuracy (described in the main text). We also describe additional components of our optimization results to help guide decisions on how to allocate sampling effort with limited resources.

Bias

We calculated relative bias, RBIAS, or the difference between our parameter estimate and the true parameter value as follows:

$$RBIAS = \frac{\left(\left({}^{1}/_{r}\right)\sum_{l=1}^{r}(\hat{\theta}_l - \theta_l)\right)}{\bar{\theta}},$$

where r was the number of replicates, $\hat{\theta}_l$ was the estimated parameter posterior median at replicate l, θ_l was the true parameter at replicate l, and $\bar{\theta}$ was the mean of the true parameter values over all replicates.

Bias with test sensitivity was most influenced by percent infected and true test sensitivity (Supplementary Figure 1). Lower test sensitivity tended to have higher negative bias with the test sensitivity parameter. Over all simulation scenarios, test sensitivity was primarily negatively biased, with less bias as the percent infected increased (Supplementary Figure 2).

Bias with prevalence was positive, and mostly influenced by the percent initially sampled and percent infected over all simulation scenarios (Supplementary Figure 3). For low prevalence (0.1%), increased sampling was more important in reducing bias than at higher prevalence levels. Using an occupancy modeling approach resulted in similar levels of bias with different test sensitivity across all simulation scenarios (Supplementary Figure 3).

Accuracy

We used relative root mean square error (RRMSE) to evaluate accuracy (the combination of bias and precision) with our simulation scenarios (defined in the main text). For test sensitivity, accuracy was highest with 10% of the population infected (Supplementary Figure 4). Accuracy of prevalence improved with increased proportions of the population initially sampled across all simulation scenarios (Supplementary Figure 5). Increased accuracy was most prominent at low prevalence (0.1%) with increases in sampling effort from the proportion of the population initially sampled, number of repeat tests, and percent of the initial population with repeat tests (Supplementary Figure 5). These improvements in accuracy with increased sampling effort were also apparent at 1% and 10% prevalence, but had smaller relative improvements in accuracy (Supplementary Figure 6).

Percent coverage

Percent coverage was calculated as the number of times the true parameter value was contained within the 95% Bayesian Credible Interval (BCI) for each replicate out of the total number of replicates. Percent coverage for test sensitivity at 10% of the population infected was high (near the nominal 95% frequentist level), but variable at lower prevalence levels (Supplementary Figure 7). Percent coverage for prevalence was low with our simulation scenarios, likely due to increases in precision for prevalence with more sampling effort but more relative bias compared to test sensitivity (Supplementary Figures 2, 3, 8, 9).

Precision

We used the 95% BCI length (upper 95% BCI minus the lower 95% BCI) to evaluate precision with our simulation scenarios. Test sensitivity and prevalence precision increased with more sampling effort, and showed similar precision between the two different test sensitivity levels as well as across the different prevalence levels (Supplementary Figures 8 and 9).

Optimization

We illustrate a constrained optimization framework over all simulation scenarios for prevalence accuracy using a constraint of $20,000. Levels of accuracy were similar for both test sensitivity values from our simulation scenarios, but costs varied greatly; thus for reduced cost, similar accuracy could be achieved with the rapid tests (Supplementary Figure 10). We also illustrate that the optimal sampling strategy is the same for 10% prevalence, compared to 1% prevalence (main text Figure 4b, Supplementary Figure 11).

References

1. Sanderlin JS, Morrison ML, Block WM. Analysis of population monitoring data. In: Brennan LA, Tri AN, Marcot BG, editors. Quantitative Analyses in Wildlife Science. Baltimore, MD: The Johns Hopkins University Press; 2019. p. 131–48.

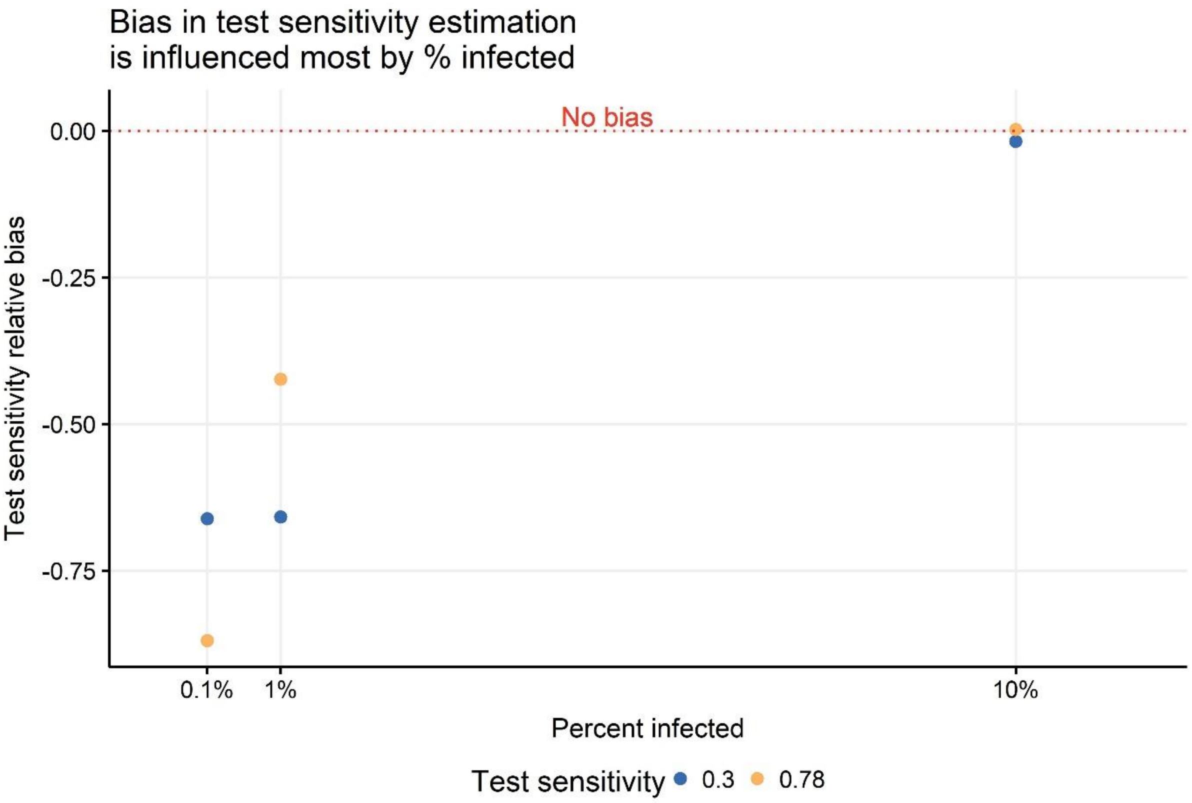

Supplementary Figure 1. Relative bias of test sensitivity as a function of percent of the population infected for simulation scenarios of 5% of the population initially sampled and 100% of that sample with 5 repeat tests.

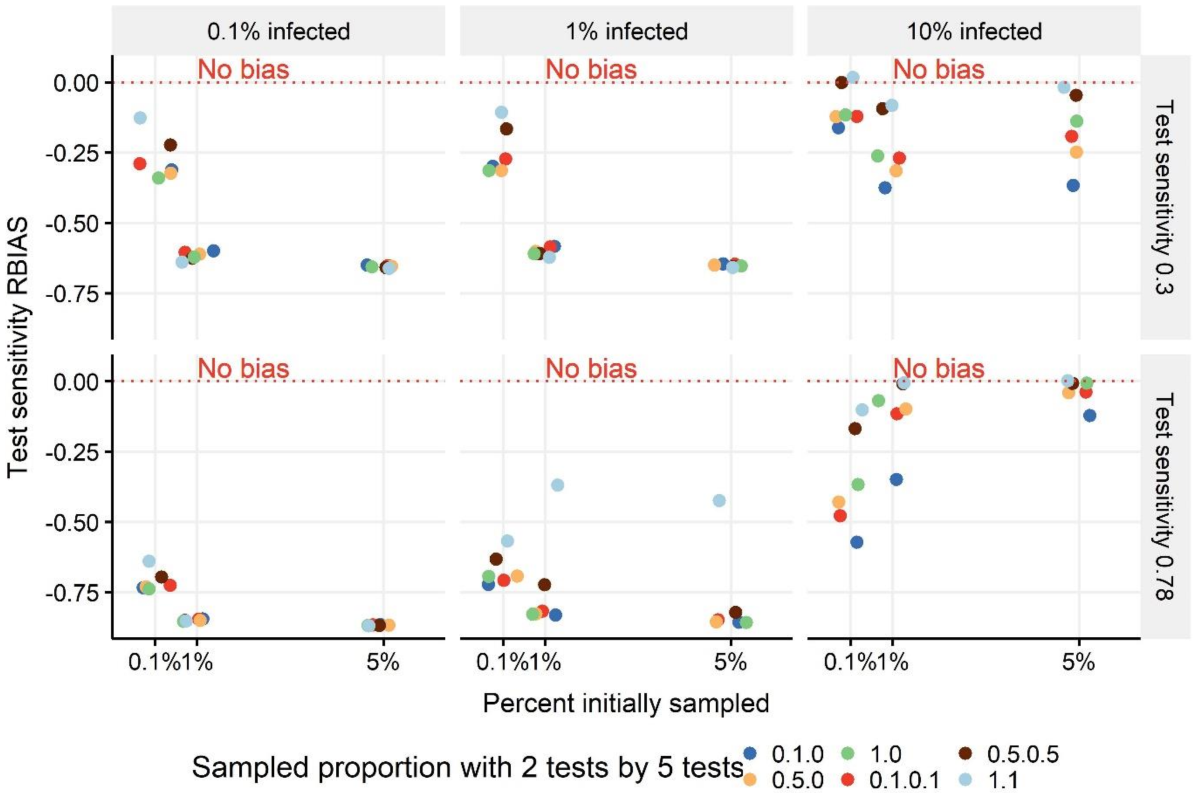

75

76 Supplementary Figure 2. Relative bias of test sensitivity as a function of percent of the
77 population infected, test sensitivity, and percent initially sampled for different sampled
78 proportions with 2 and 5 repeat tests.

79

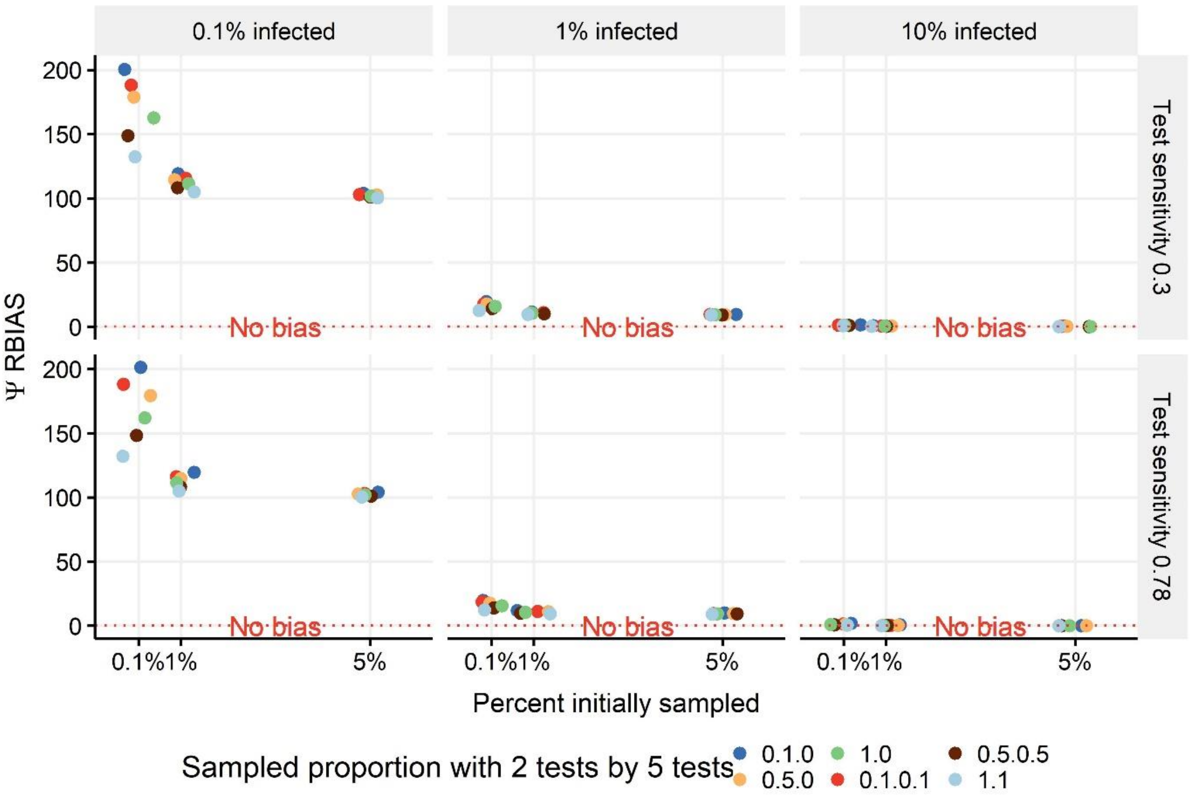

Supplementary Figure 3. Relative bias of prevalence as a function of percent of the population infected, test sensitivity, and percent initially sampled for different sampled proportions with 2 and 5 repeat tests.

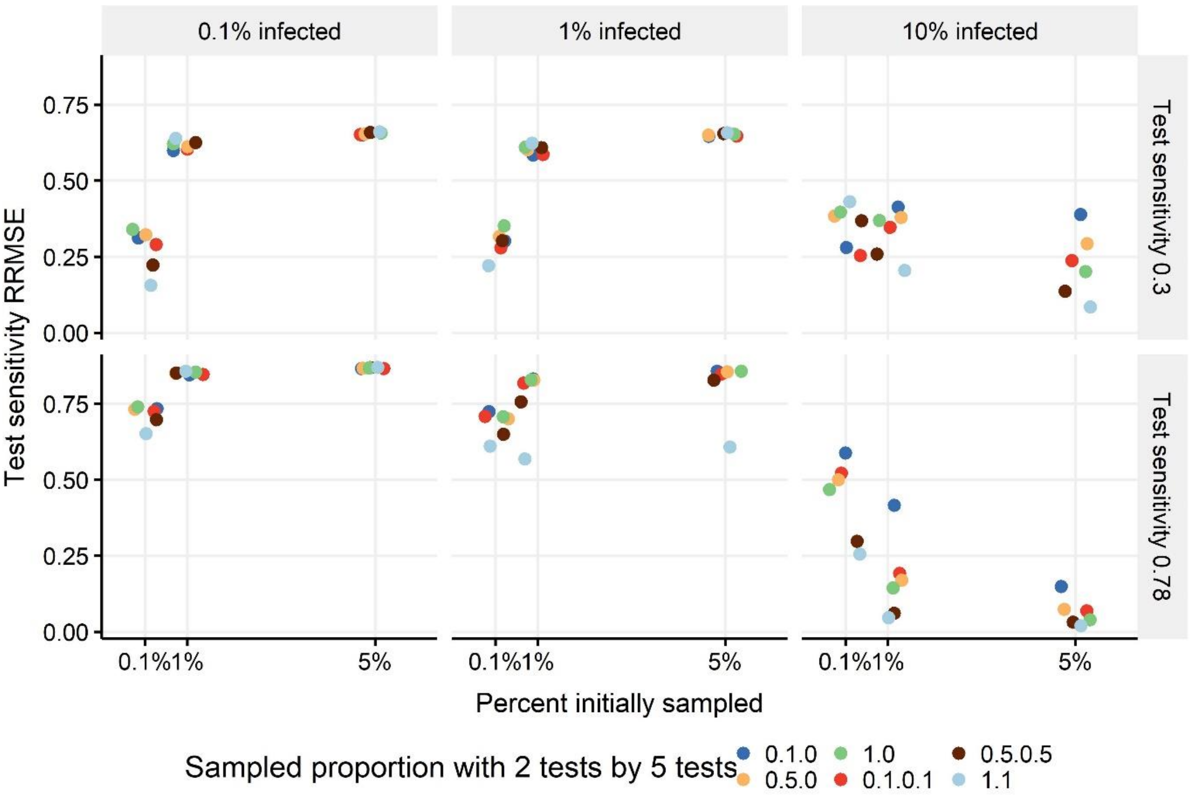

Supplementary Figure 4. Accuracy (Relative root mean square error; RRMSE) of test sensitivity as a function of percent of the population infected, test sensitivity, and percent initially sampled for different sampled proportions with 2 and 5 repeat tests.

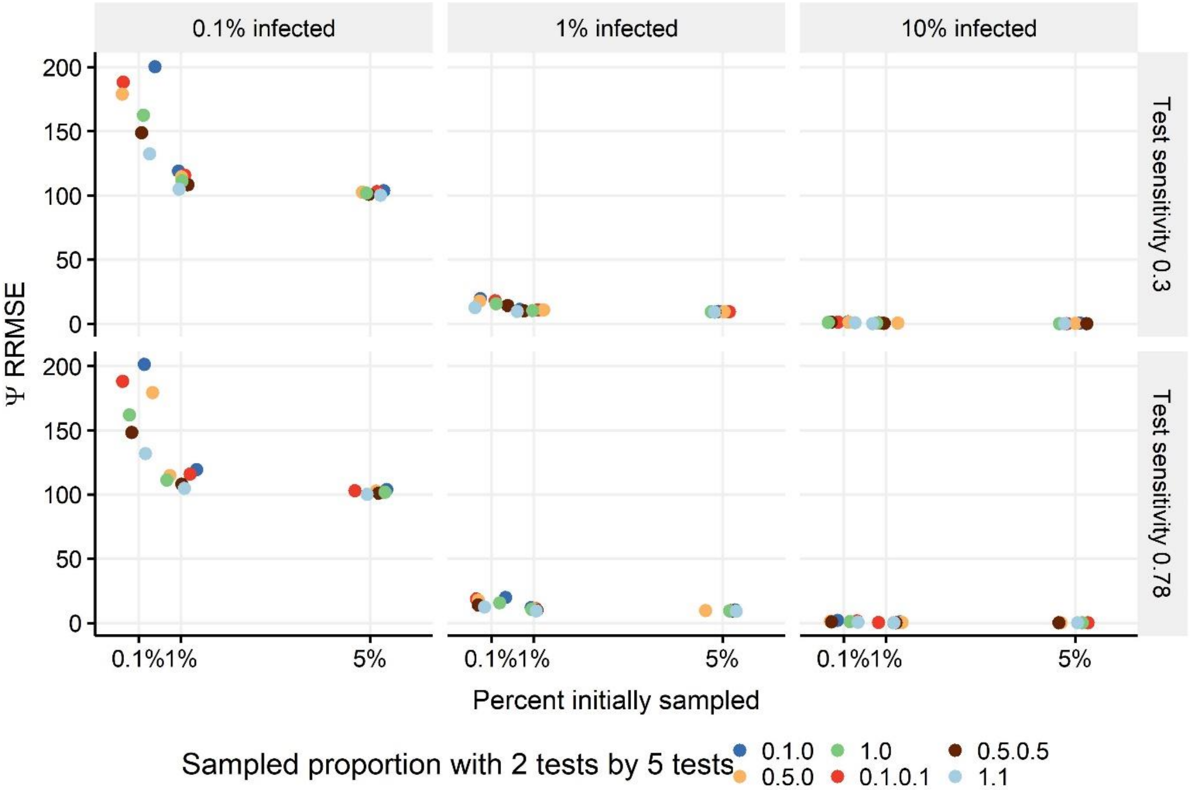

Supplementary Figure 5. Accuracy (Relative root mean square error; RRMSE) of prevalence as a function of percent of the population infected, test sensitivity, and percent initially sampled for different sampled proportions with 2 and 5 repeat tests.

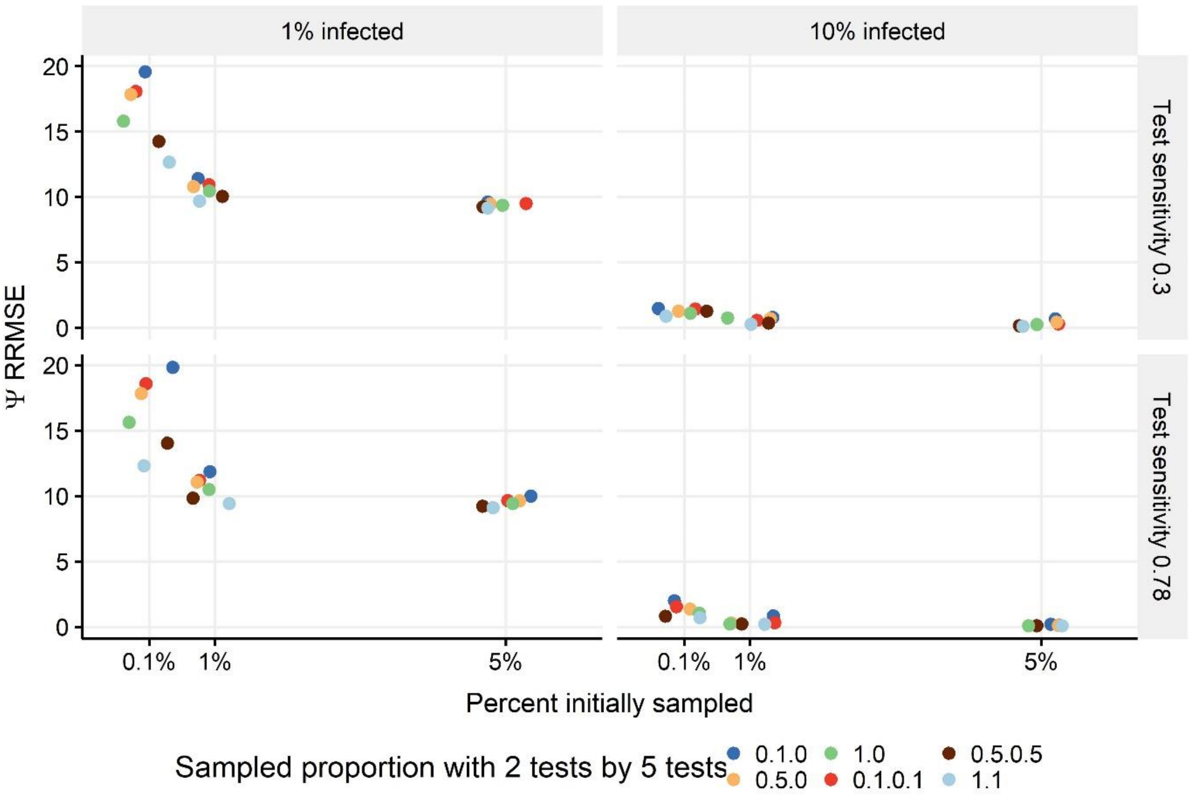

Supplementary Figure 6. Accuracy (Relative root mean square error; RRMSE) of prevalence as a function of 1% and 10% of the population infected, test sensitivity, and percent initially sampled for different sampled proportions with 2 and 5 repeat tests.

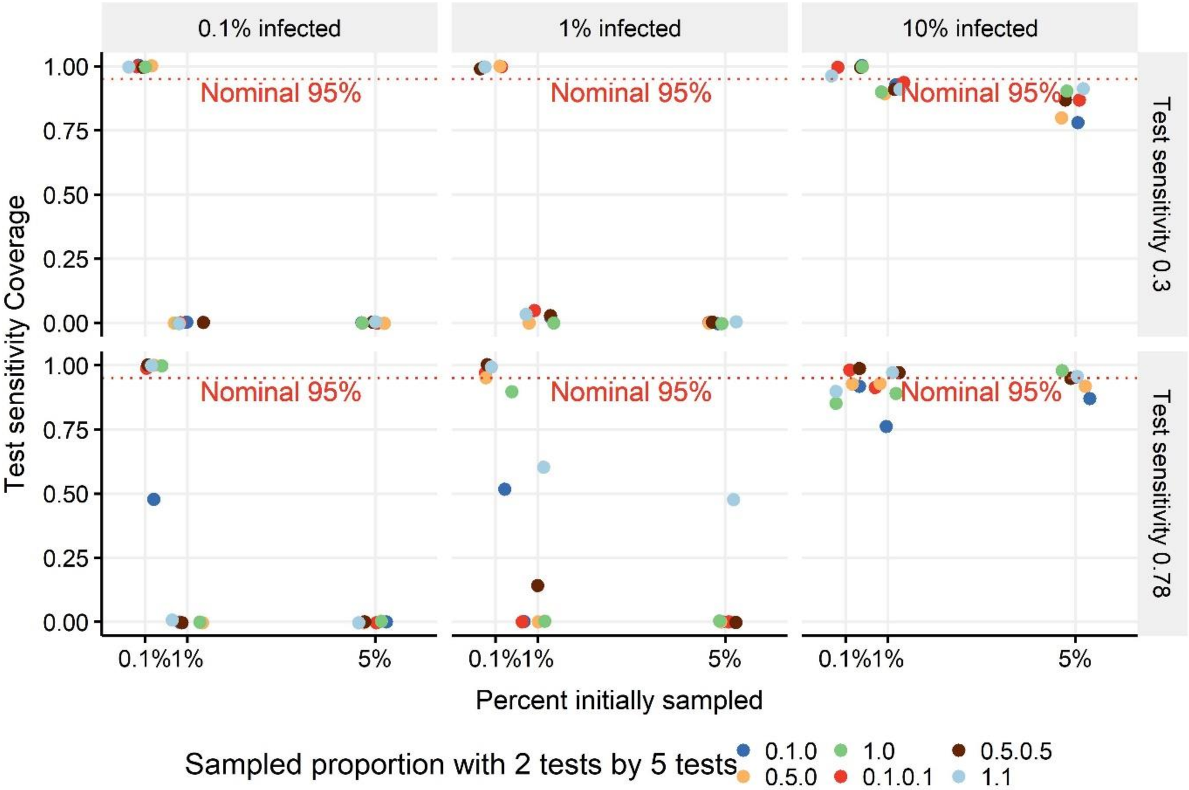

Supplementary Figure 7. Percent coverage of test sensitivity as a function of percent of the population infected, test sensitivity, and percent initially sampled for different sampled proportions with 2 and 5 repeat tests. Nominal 95% frequentist level for coverage is indicated on the plot.

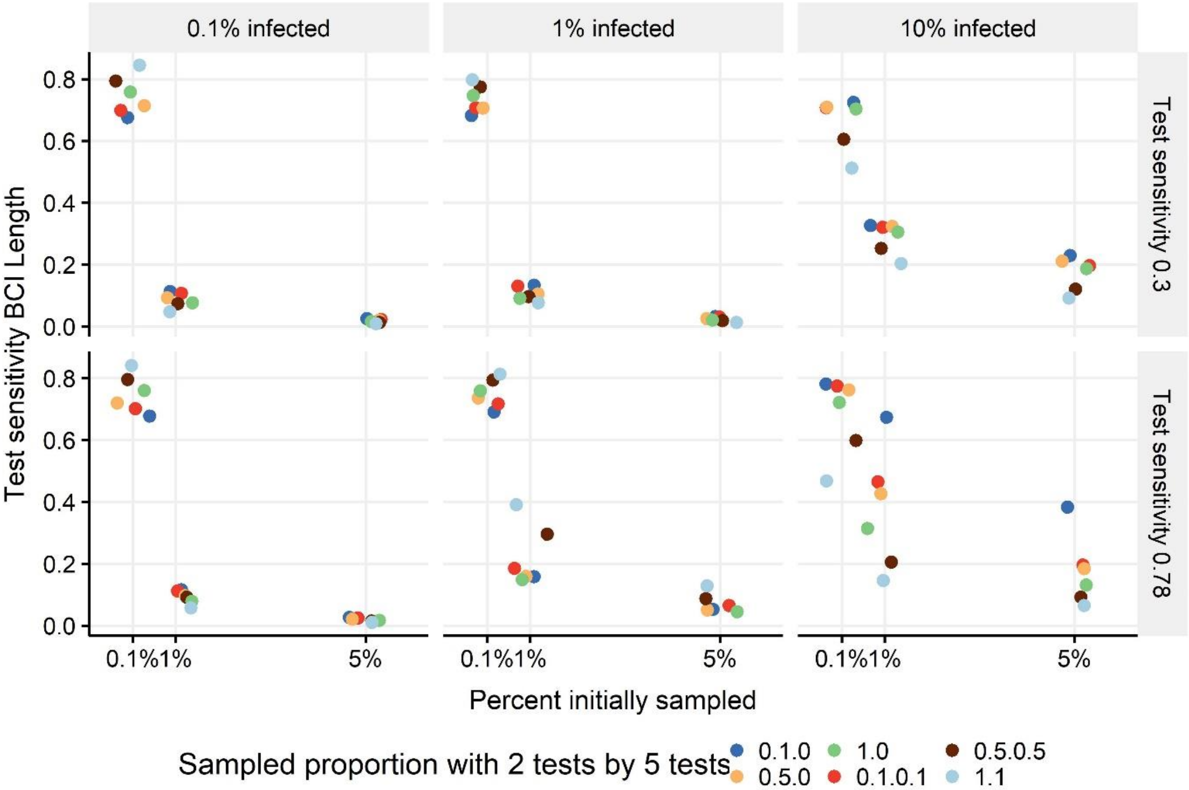

Supplementary Figure 8. Precision (Bayesian Credible Interval length; BCI length) of test sensitivity as a function of percent of the population infected, test sensitivity, and percent initially sampled for different sampled proportions with 2 and 5 repeat tests.

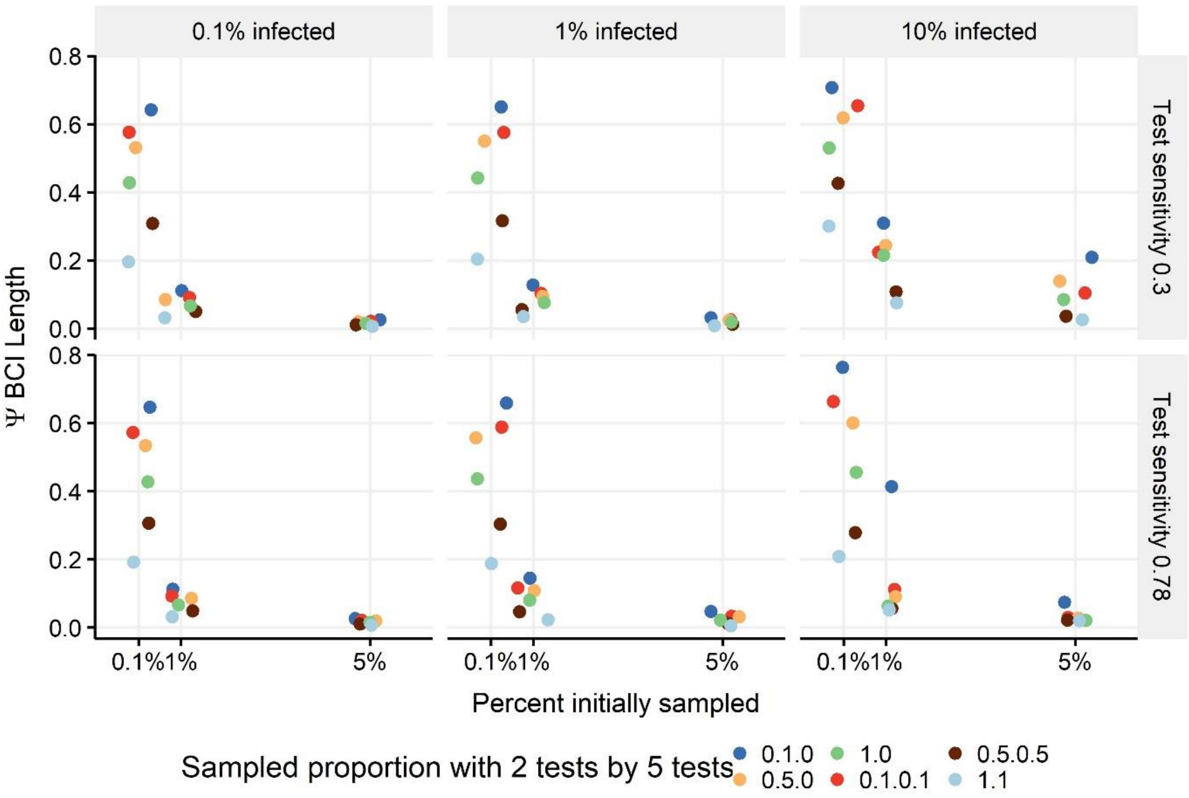

Supplementary Figure 9. Precision (Bayesian Credible Interval length; BCI length) of prevalence as a function of percent of the population infected, test sensitivity, and percent initially sampled for different sampled proportions with 2 and 5 repeat tests.

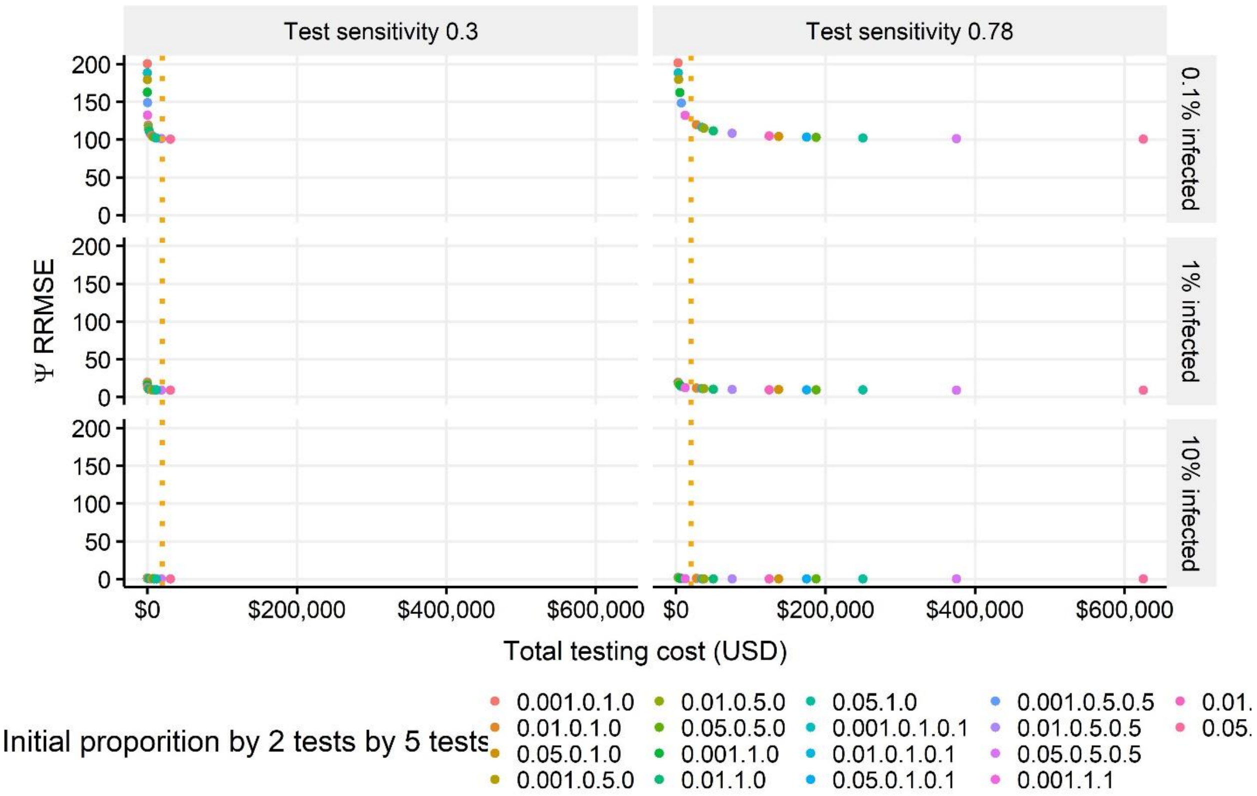

Supplementary Figure 10. Accuracy (relative root mean square error) of prevalence (*Ψ*) as a function of costs (USD) using arbitrary cost constraint (dotted vertical line) of $20,000 USD for all simulation scenarios as a function of percent of the population infected and test sensitivity for different sampled proportions of the population initially sampled, with 2 repeat tests, and with 5 repeat tests.

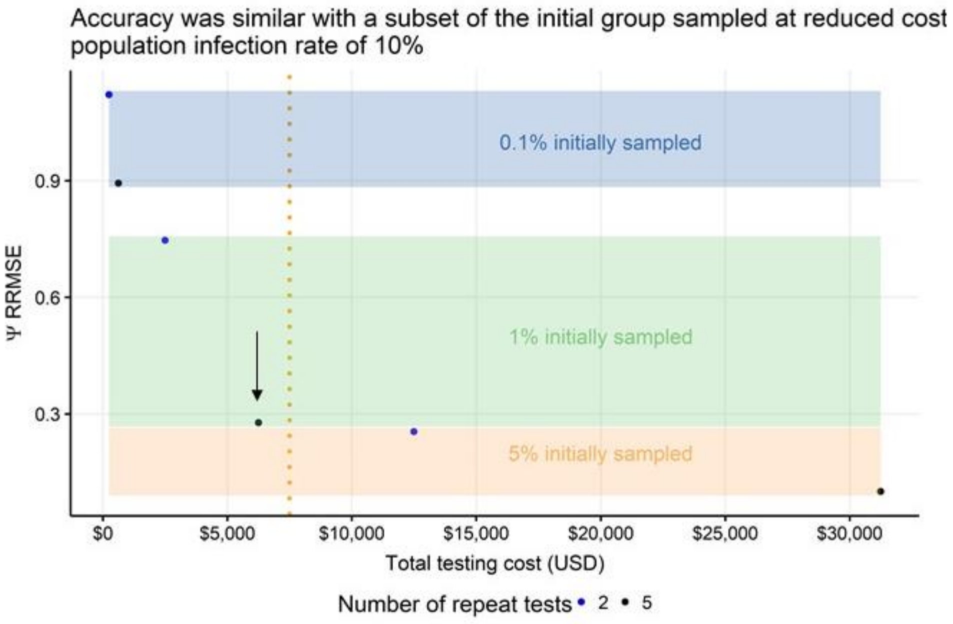

Supplementary Figure 11. Accuracy (relative root mean square error) of prevalence (Ψ) as a function of costs (USD) using arbitrary cost constraint (dotted vertical line) of $7,500 USD for a subset of the simulation scenarios with the true test sensitivity of 0.3, true population infection rate (true prevalence) of 10%, and 100% of the initially sampled population with repeat tests. Optimal design is indicated within the figure with an arrow.
