# Supplementary material for: Occupancy modeling and resampling overcomes low test sensitivity to produce accurate SARS-CoV-2 prevalence estimates
Source: BMC Public Health. 2021 Mar 23;21:577. doi: 10.1186/s12889-021-10609-y (PMC7986646; doi:10.1186/s12889-021-10609-y)
Supplement: Supplementary file 3 — Additional file 3. Simulation code. R code of simulations (GitHub: https://github.com/jamiesanderlin/Sanderlin-et-al-occupancy-modeling-and-SARS-CoV-2-prevalence). [file 12889_2021_10609_MOESM3_ESM.pdf]

Additional file 3. Simulation code. Computer code used to model SARS-CoV-2 surveys in program R (1) with JAGS (2), *rjags*, *jagsUI* (3), and *coda* (4).

This section of code is contained in the script "covid_load_packages.R" and is used to install and load the packages required to run simulations.

```
# COVID model
# Code purpose: create simple model for COVID simulation with imperfect detection
# Load packages (check if installed and if not install them)
#################################################################################
## Load required packages
# Function from https://vbaliga.github.io/verify-that-r-packages-are-installed-and-loaded/
## First specify the packages of interest
packages = c("jagsUI", "mcmcplots","reshape2","here",
             "dplyr", "ggplot2","psych", "extraDistr")

## Now load or install&load all
package.check <- lapply(
  packages,
  FUN = function(x) {
    if (!require(x, character.only = TRUE)) {
```

```
      install.packages(x, dependencies = TRUE)
      library(x, character.only = TRUE)
    }
  }
)

This section of code is contained in the script "covid_sc1_sim_bio.R" and is used to model the
biological conditions used for simulations.
### Single-season occupancy category model to count covid infected individuals
# Function
####################################### Intro #######################################
# Name: sc1_sim_bio
# Description:  function to simulate biological conditions of a population at
#           multiple sites with two states of people at a site (infected and
#           not infected), governed by a given prevalence of covid infection
################################### Arguments ###################################
# n.counties:
#     Number of counties. Whole number.
#     Manuscript terminology: County
```

```
# mu.pop:
#     Number of people per county. Whole number.
#     Manuscript terminology: Average county population
# psi.infected:
#     Average occupancy of the covid infection per county. Between 0 and 1.
#     Manuscript terminology: Probability of being infected

################################### Output ######################################
# biodata list saved to global environment. Contains n.counties, mu.pop, psi.infected,
# infected, uninfected, and z
#################################################################################
sc1_sim_bio <- function(n.counties, mu.pop, psi.infected){

  # Total population size each location
  population <- rep(mu.pop, n.counties)

  # Infection status per individual
  z <- matrix(data=NA, nrow = mu.pop, ncol = n.counties)
```

```
57      for(i in 1:mu.pop){
58        for (j in 1:n.counties){
59          z[i,j] <- rbinom(1,1,psi.infected)
60        }
61      }
62
63      # Number of uninfected people per location
64      uninfect <- c()
65      infect <- c()
66      for (i in 1:n.counties){
67        uninfect[i] <-population[i]-sum(z[,i])
68        infect[i] <-sum(z[,i])
69      }
70      biodata <- list("n.counties"= n.counties, "mu.pop"=mu.pop,"population"=population,
71                      "z"= z, "uninfect" = uninfect, "infect"= infect, "psi.infected"=psi.infected)
72      list2env(biodata ,.GlobalEnv)
73    }
74
```

This section of code is contained in the script “covid_sc1_sim_obs.R” and is used to model the observational conditions used for simulations.

```
### Single-season occupancy category model to count covid infected individuals
##################################### Intro #####################################
# Name: sc1_sim_obs
# Description:  function to simulate observation  of a population at multiple sites
#               with two states of people at a site (infected and not infected)
#               and repeat sampling events with a known number of samples taken
#               at each event
################################## Arguments ##################################
# z:
#     Occupancy of covid virus at each person. 0 or 1.
#     Manuscript terminology: psi subscript I
# mu.pop:
#     Number of people per county. Whole number.
#     Manuscript terminology: Average county population
# n.counties:
#     Number of counties. Whole number.
#     Manuscript terminology: County
```

```
# p.test:
#     Test sensitivity. Between 0 and 1.
#     Manuscript terminology: Test sensitivity
# p.init.samp:
#     Proportion of county intially sampled. Between 0 and 1.
#     Manuscript terminology: Proportion of sampled individuals
# p.rep1.samp:
#     Proportion of people intially sampled that are resampled in repeat 1. Between 0 and 1.
#     Manuscript terminology: Proportion of sampled individuals with repeat swabs
# p.rep2.samp:
#     Proportion of people intially sampled that are resampled in repeat 2. Between 0 and 1.
#     Manuscript terminology: Proportion of sampled individuals with repeat swabs
# p.rep3.samp:
#     Proportion of people intially sampled that are resampled in repeat 3. Between 0 and 1.
#     Manuscript terminology: Proportion of sampled individuals with repeat swabs
# p.rep4.samp:
#     Proportion of people intially sampled that are resampled in repeat 4. Between 0 and 1.
#     Manuscript terminology: Proportion of sampled individuals with repeat swabs
```

```
112
113  ################################### Output ######################################
114
115  # obsdata list saved to global environment. Contains p.test, y1, yselect, p.eff,
116  # n.obs, site (index value for site), event (index value for event), swab (index
117  # value for swab), n.events, n.swabs, n.init.samp (number of initial samples per county),
118  # n.rep1.samp (number of repeat samples per county),
119  # n.rep2.samp (number of second repeat samples per county)
120
121  ################################################################################
122  sc1_sim_obs <- function(z, p.test, n.counties, p.init.samp, p.rep1.samp, p.rep2.samp,
123  p.rep3.samp, p.rep4.samp){
124
125    # Effective detection probability
126    p.eff <-z*p.test
127
128    #Create indexing values
129    n.init.samp = round(mu.pop*p.init.samp)
130    n.rep1.samp = round(n.init.samp*p.rep1.samp)
```

```
n.rep2.samp = round(n.init.samp*p.rep2.samp)
n.rep3.samp = round(n.init.samp*p.rep3.samp)
n.rep4.samp = round(n.init.samp*p.rep4.samp)
n.obs <-mu.pop*n.counties
county <-rep(1:n.counties, each = mu.pop)
init.samp <-rep(c((rep(1, n.init.samp)), (rep(NA,mu.pop-n.init.samp))),n.counties)
rep1.samp <-rep(c((rep(1, n.rep1.samp)), (rep(NA,mu.pop-n.rep1.samp))),n.counties)
rep2.samp <-rep(c((rep(1, n.rep2.samp)), (rep(NA,mu.pop-n.rep2.samp))),n.counties)
rep3.samp <-rep(c((rep(1, n.rep3.samp)), (rep(NA,mu.pop-n.rep3.samp))),n.counties)
rep4.samp <-rep(c((rep(1, n.rep4.samp)), (rep(NA,mu.pop-n.rep4.samp))),n.counties)

y1<-matrix(data=NA, nrow = mu.pop*n.counties, ncol = 12)
colnames(y1)<-
c("county","init.samp","detect.init","rep1.samp","detect.rep1","rep2.samp","detect.rep2","rep3.sa
mp","detect.rep3","rep4.samp","detect.rep4","person")
y1[,1] <-as.vector(county)
y1[,2] <-as.vector(init.samp)
y1[,4] <-as.vector(rep1.samp)
y1[,6] <-as.vector(rep2.samp)
```

```
150     y1[,8] <-as.vector(rep3.samp)
151     y1[,10] <-as.vector(rep4.samp)
152     y1[,12] <-as.vector(replicate(n.counties, sample(1:mu.pop,mu.pop,replace=FALSE)))
153
154     for(i in 1:n.obs){
155       y1[i,3] <- rbinom(1,1,p.eff[y1[i,12],county[i]])*y1[i,2]
156       y1[i,5] <- rbinom(1,1,p.eff[y1[i,12],county[i]])*y1[i,4]
157       y1[i,7] <- rbinom(1,1,p.eff[y1[i,12],county[i]])*y1[i,6]
158       y1[i,9] <- rbinom(1,1,p.eff[y1[i,12],county[i]])*y1[i,8]
159       y1[i,11] <- rbinom(1,1,p.eff[y1[i,12],county[i]])*y1[i,10]
160     }
161
162     obsdata <- list("p.test"= p.test, "y1"= y1, "p.eff"= p.eff,
163                     "n.obs"= n.obs, "county" = county,
164                     "p.init.samp" = p.init.samp,"p.rep1.samp" = p.rep1.samp,
165                     "p.rep2.samp" = p.rep2.samp,"p.rep3.samp" = p.rep3.samp,
166                     "p.rep4.samp" = p.rep4.samp,"n.init.samp" = n.init.samp,
167                     "n.rep1.samp"= n.rep1.samp,"n.rep2.samp"= n.rep2.samp,
```

```
                "n.rep3.samp"= n.rep3.samp,"n.rep4.samp"= n.rep4.samp)
  list2env(obsdata ,.GlobalEnv)
}
```

This section of code is contained in the script “covid_sc1_run.R” and is used to create a Bayesian hierarchical model of biological conditions in simulated scenarios.

```
### Single-season occupancy category model to count covid infected individuals
##################################### Intro ###################################
# Name: sc1_run
# Description:  function to run Bayesian hierarchical model of biological
#           conditions of a population at multiple sites with two states of
#           people at a site (infected and not infected), governed by a
#           given prevalence of covid infection (sc1_sim_bio).
################################## Arguments ##################################
# data:
#     List of data to use for model.
# inits:
#     Initial values for z (occupancy of virus).
# params:
```

```
#       Parameters to keep track of.
# ni:
#       Number of iterations to run the Bayesian model.
# nt:
#       Thinning parameter for JAGS.
# nb:
#       Number of burn in iterations.
# nc:
#       Number of MCMC chains to run.
# na:
#       Adapt parameter - number of adaptive iterations to start the simulation .

################################### Output ######################################
# out, a large jags list object saved to global environment
#################################################################################
sc1_run <- function(data, inits, params, ni, nt, nb, nc, na){

  ### JAGS MODEL
```

```
sink("Model.txt")
cat("
  model{
  # Priors
  # inf
  psi_infected ~ dunif(0.1, 1)

  # test sensitivity
  p ~ dunif(0.1, 1)

  for(i in 1:mu.pop){
  for (j in 1:n.counties){
    # Biological model
    z[i,j] ~ dbern(psi_infected)

    # Detection model - random sample
    p.eff[i,j] <-z[i,j] * p
  }
```

```
223       }
224
225       # Derived abundance values in each class
226       for (i in 1:n.counties){
227         uninfect[i] <-population[i]-sum(z[,i])
228         infect[i] <-sum(z[,i])
229       }
230
231       for (i in 1:n.obs){
232         y1[i,3] ~ dbern(p.eff[y1[i,12],county[i]])
233         y1[i,5] ~ dbern(p.eff[y1[i,12],county[i]])
234         y1[i,7] ~ dbern(p.eff[y1[i,12],county[i]])
235         y1[i,9] ~ dbern(p.eff[y1[i,12],county[i]])
236         y1[i,11] ~ dbern(p.eff[y1[i,12],county[i]])
237       }
238       }
239       ", fill = TRUE)
240     sink()
```

```
241
242   out<- list(jags(data=data, inits=inits, parameters.to.save = params, "Model.txt",
243                   n.chains=nc,n.thin=nt, n.iter=ni, n.burnin=nb, n.adapt=na,
244                   parallel = TRUE))
245   out
246 }
247
248 This section of code is contained in the script “covid_sc1_sim_posi.R” and is used to calculate
249 the percentage of tests with a positive SARS-CoV-2 result from the observed samples.
250 ### Single-season occupancy category model to count covid infected individuals
251 ######################################## Intro ########################################
252 # Name: sc1_posi
253 # Description:  function to calculate percent positive out of all tests from the observed samples.
254 # This will provide a comparison to the reported measure of percent positive that is used to
255 # make decisions, which is not individual based (i.e., repeated tests of individuals are not
256 # accounted for within the observed reported measure, which is also not accounting for
257 # uncertainty in testing)
258
259 ################################### Arguments ###################################
```

```
# data:
#       List of data to use for calculation of percent positive in observed samples.

sc1_sim_posi <- function(data){

  p.pos <-
(sum(y1[1:n.init.samp,3],y1[1:n.rep1.samp,5],y1[1:n.rep2.samp,7],y1[1:n.rep3.samp,9],y1[1:n.re
p4.samp,11],na.rm=TRUE))/(sum(y1[1:n.init.samp,2],y1[1:n.rep1.samp,4],y1[1:n.rep2.samp,6],
y1[1:n.rep3.samp,8],y1[1:n.rep2.samp,10],na.rm=TRUE))

  p.pos
}
```

This section of code is contained in the script “covid_sc2_execute.R” and is an example of code used to run an occupancy model of a simulated SARS-CoV-2 sampling scenario. This code was subsequently modified to produce each of the 108 scenarios examined.

```
# COVID model

# Code purpose: create simple file for running COVID simulations
```

```
279
280 ##############################################################################
281 ## Load functions
282 ############## UPDATE WITH APPROPRIATE DIRECTORY FOR YOUR COMPUTER ##
283 source("your/path/here/covid_load_packages.R")
284 source("your/path/here/covid_sc1_sim_bio.R")
285 source("your/path/here/covid_sc1_sim_obs.R")
286 source("your/path/here/covid_sc1_run.R")
287 source("your/path/here/covid_sc1_sim_posi.R")
288
289 ### Repitition of simulation
290 # Specify number of reps
291 n.reps <-100
292
293 # Create empty list to hold rep info
294 fit <- list()
295
296 for(q in 1:n.reps){
```

```
297     # Time each rep
298     ptm <- proc.time()
299
300     ## Simulate Data
301     # Biological process
302     sc1_sim_bio(n.counties = 1, mu.pop = 25000, psi.infected = 0.001)
303
304     # Observation process
305     sc1_sim_obs(z = z, p.test = 0.3, n.counties = n.counties, p.init.samp = 0.001, p.rep1.samp = 0.5,
306   p.rep2.samp = 0.5, p.rep3.samp = 0.5, p.rep4.samp = 0.5)
307
308     ## List data for running model
309     # Main data
310     data <- list(y1=y1,
311                  mu.pop = mu.pop,
312                  population = population,
313                  n.counties = n.counties,
314                  n.obs = n.obs,
315                  county = county,
```

```
316             n.init.samp = n.init.samp,
317             n.rep1.samp = n.rep1.samp,
318             n.rep2.samp = n.rep2.samp,
319             n.rep3.samp = n.rep3.samp,
320             n.rep4.samp = n.rep4.samp)
321
322     #calculate observed percent positive
323     per.positive <- sc1_sim_posi(data)
324
325     # Initial value data
326     inits <- function() {
327       list(z = z)
328     }
329
330     # Parameters for the model to keep track of
331     params <- c("psi_infected", "p","infect","uninfect")
332
333     ## Run model
```

```
334     out <-sc1_run(data = data, inits = inits, params = params, ni= 10000, nt = 1, nb = 1000, nc = 3,
335   na = 1000)
336     #add observed percent positive to output
337     finalout <- c(out,per.positive)
338     time <-proc.time() - ptm
339     name <-paste("rep",q,"_",n.counties,"county", "_", mu.pop, "pop",
340                  "_", psi.infected,"psi", "_", p.test,"ptest",
341                  "_", ifelse(p.init.samp*100<100&p.init.samp*100>9,paste(0,p.init.samp*100,sep=""),
342
343   ifelse(p.init.samp*100<10,paste(0,0,p.init.samp*100,sep=""),p.init.samp*100)),"psamp",
344                  "_",
345   ifelse(p.rep1.samp*100<100&p.rep1.samp*100>9,paste(0,p.rep1.samp*100,sep=""),
346
347   ifelse(p.rep1.samp*100<10,paste(0,0,p.rep1.samp*100,sep=""),p.rep1.samp*100)), "pr1",
348                  "_",
349   ifelse(p.rep2.samp*100<100&p.rep2.samp*100>9,paste(0,p.rep2.samp*100,sep=""),
350
351   ifelse(p.rep2.samp*100<10,paste(0,0,p.rep2.samp*100,sep=""),p.rep2.samp*100)),"pr2",
352                  "_",
353   ifelse(p.rep3.samp*100<100&p.rep3.samp*100>9,paste(0,p.rep3.samp*100,sep=""),
```

```
ifelse(p.rep3.samp*100<10,paste(0,0,p.rep3.samp*100,sep=""),p.rep3.samp*100)),"pr3",
                "_",
ifelse(p.rep4.samp*100<100&p.rep4.samp*100>9,paste(0,p.rep4.samp*100,sep=""),

ifelse(p.rep4.samp*100<10,paste(0,0,p.rep4.samp*100,sep=""),p.rep4.samp*100)),"pr4",
                sep="")

  ############## UPDATE WITH APPROPRIATE DIRECTORY FOR YOUR COMPUTER##
  #save(finalout,file= paste0 ("your/path/here/", name))
  save(finalout,file= paste0 ("your/path/here/", name))

  fit[[q]]<-list(name, finalout, time)
}
```

References

1. R Core Team. R: a language and environment for statistical computing [Internet]. Vienna, Austria: R Foundation for Statistical Computing; 2020. Available from: https://www.r-project.org

2. Plummer M. JAGS: A program for analysis of Bayesian graphical models using Gibbs sampling. Proc 3rd Int Work Distrib Stat Comput (DSC 2003). 2003;20–2.

3. Kellner K. jagsU: a wrapper around “jags” to streamline ‘JAGS’ analyses [Internet]. jR package version 1.4.2. https://CRAN.R-project.org/package=jagsUI’, jagsUI: A Wrapper Around ‘rjags’ to Streamline ‘JAGS’ Analyses. R package version 1.4.2. https://CRAN.R-project.org/package=jagsUI. 2016. Available from: https://cran.r-project.org/web/ packages/jagsUI/index.html

4. Plummer M, Best N, Cowles K, Vines K. CODA: Convergence diagnosis and output analysis for MCMC. R News [Internet]. 2006;6(1):7–11. Available from: https://cran.r-project.org/web/ packages/coda/index.html
