# Supplementary material for: Occupancy modeling and resampling overcomes low test sensitivity to produce accurate SARS-CoV-2 prevalence estimates
Source: BMC Public Health. 2021 Mar 23;21:577. doi: 10.1186/s12889-021-10609-y (PMC7986646; doi:10.1186/s12889-021-10609-y)
Supplement: Supplementary file 5 — Additional file 5. Individual-level inference from occupancy model results. Document describing individual-level inference from occupancy model results. [file 12889_2021_10609_MOESM5_ESM.pdf]

Additional file 5. Individual-level inference from occupancy model results

In occupancy modeling, wildlife and fisheries literature recommends sampling more sites (individuals) fewer times (repeat tests) as preferable when species are particularly rare (1–3). General sampling recommendations also include increasing the number of sites instead of sampling occasions per site when detection probabilities are high and increasing sampling occasions per site as detection probabilities decrease (4). These sampling recommendations are recommended for prevalence inference but can also assist with individual-level inference due to better estimates of test sensitivity using repeated samples necessary for occupancy modeling.

Individual-level inference requires accurate estimates of test sensitivity and may have different optimal sampling strategies if the objective is at the individual-level due to false negatives with testing (Supplementary Figure 1). We illustrate this concept with the number of repeat tests needed for determining the probability of detecting the disease at the individual level using our true test sensitivity values of 0.3 and 0.78. The probability of a false negative is calculated as: $(1 - \textit{test sensitivity})^k$ where k is the number of tests. From the occupancy literature(5) a range of 0.05 to 0.15 is recommended for best inference for the probability of a false negative. The probability of detecting the disease at least once during k tests with an individual that has the disease (p^* in the occupancy literature) is calculated as: $1 - (1 - \textit{test sensitivity})^k$. Using our true values for test sensitivity, we illustrate how calculating the probability of detecting the disease at least once during k tests with an individual that has the disease shows that repeat sampling is more important with lower test sensitivities (Supplementary Figure 2).

References

1. Sanderlin JS, Block WM, Ganey JL. Optimizing study design for multi-species avian monitoring programmes. J Appl Ecol. 2014;51(4):860–70.

2. Mackenzie DI, Royle JA. Designing Occupancy Studies : General Advice and Allocating Survey Effort. J Appl Ecol. 2005;42(6):1105–14.

3. Field S, Tyre A, Possingham H. Optimizing Allocation of Monitoring Effort Under Economic and Observational Constraints. J Wildl Manage. 2005;69(3):473–82.

4. Tyre AJ, Tenhumberg B, Field SA, Niejalke D, Parris K, Possingham HP. Improving precision and reducing bias in biological surveys: Estimating false-negative error rates. Ecol Appl. 2003;13(6):1790–801.

5. Mackenzie DI, Nichols JD, Royle JA, Pollock KH, Bailey LL, Hines JE. Occupancy estimation and modeling. New York, New York: Academic Press; 2006.

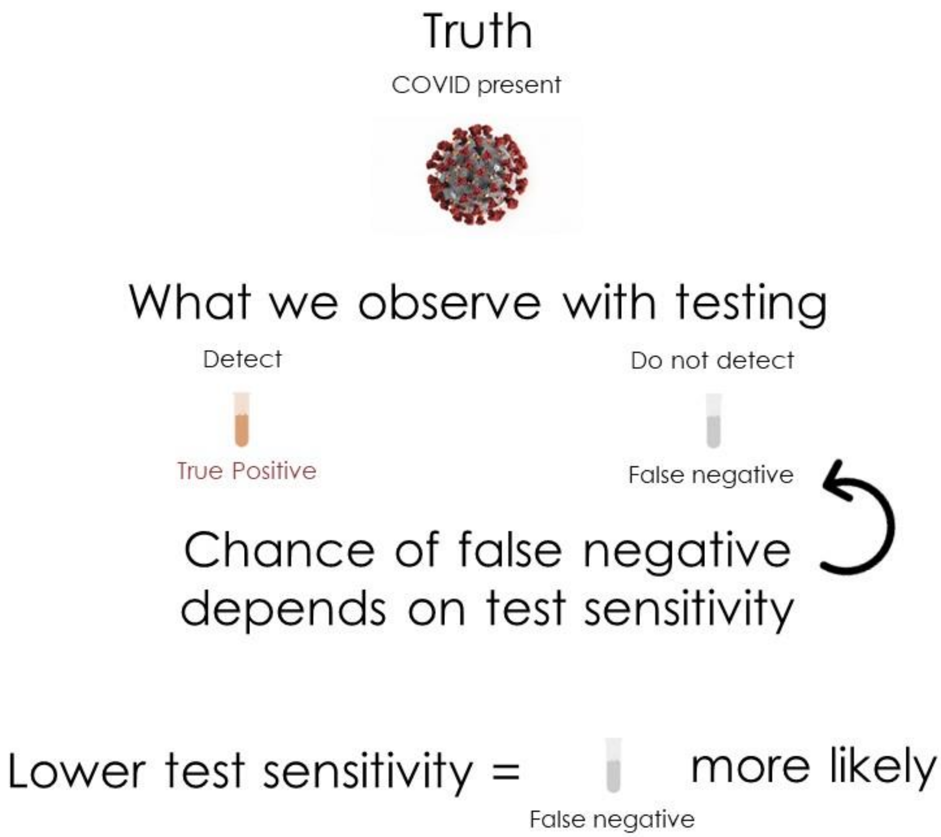

Supplementary Figure 1. Probability of a false negative and test sensitivity. SARS-CoV-2 image credit: Centers for Disease Control and Prevention, Alissa Eckert (MSMI) and Dan Higgins (MAMS).

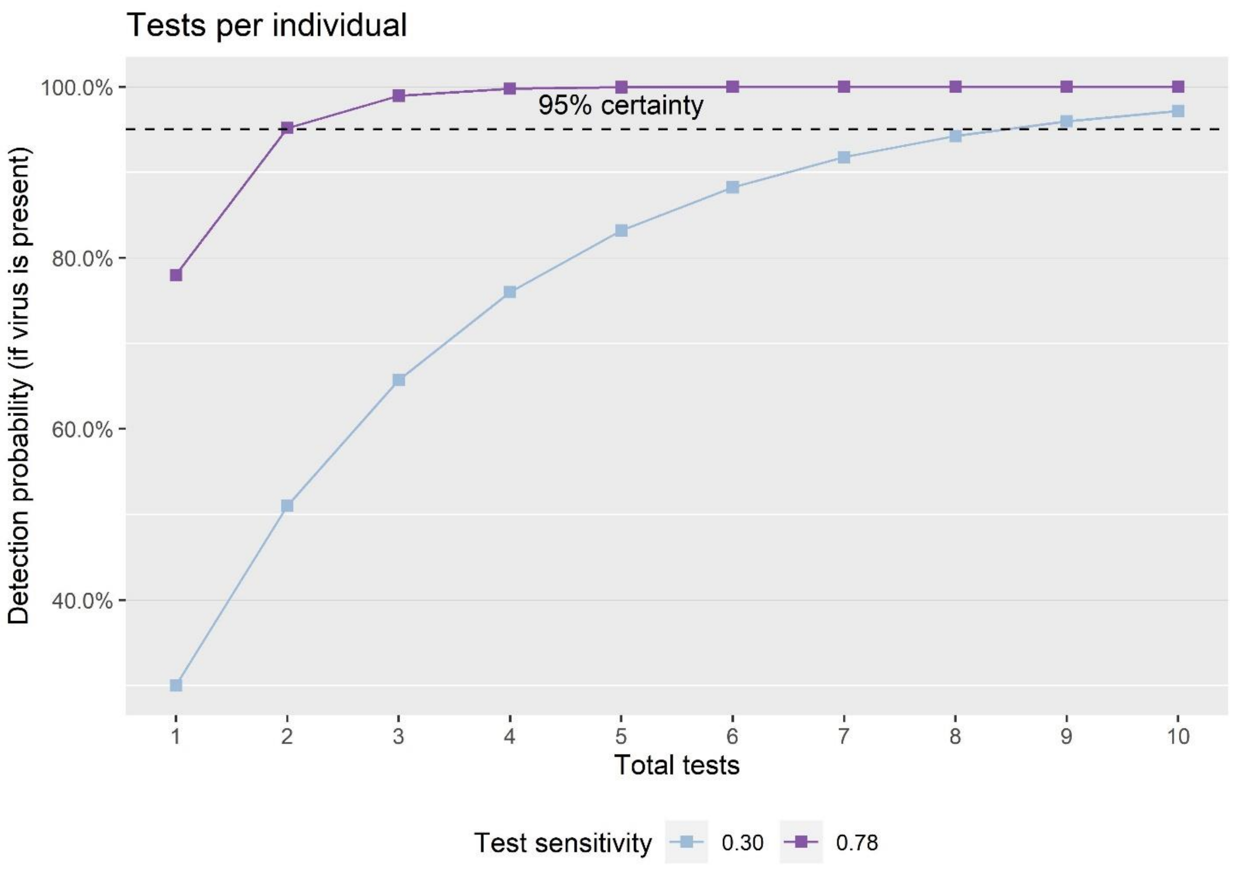

Supplementary Figure 2. Probability of detecting the disease at least once with an individual that has the disease as a function of total tests. Occupancy literature recommends a false negative error rate range between 0.05-0.15 (or a 95% probability of detecting the disease at least once).
